# Proteomics- and BRET- screens identify SPRY2 as a Ras effector that impacts its membrane organization

**DOI:** 10.1016/j.isci.2025.113974

**Published:** 2025-11-07

**Authors:** Karolina Pavic, Fiona Elizabeth Hood, Carla Jane Duval, Ganesh babu Manoharan, Christina Laurini, Farid Ahmad Siddiqui, Stephanie Puy Lam Mo, Ian Andrew Prior, Daniel Kwaku Abankwa

**Affiliations:** 1 Cancer Cell Biology and Drug Discovery Group, Department of Life Sciences and Medicine, University of Luxembourg, 4362 Esch-sur-Alzette, Luxembourg; 2 Department of Molecular and Clinical Cancer Medicine, Institute of Systems, Molecular and Integrative Biology, University of Liverpool, Liverpool L69 3BX, UK; 3 Institute of Biomedicine, University of Turku, 20520 Turku, Finland

**Keywords:** Molecular biology, Membrane architecture, Proteomics

## Abstract

Few regulators of K-Ras plasma membrane organization are known. We combined TurboID-based proximity proteomics with a BRET screen to identify eight potential K-Ras G-domain interactors. We focused on APLP2, which indirectly engages with C-Raf in immediate proximity to K-Ras, and SPRY2, which exhibits properties of an effector. Co-immunoprecipitation and BRET assays revealed that the SPRY2 C-terminal half binds oncogenic RasG12V more than full-length SPRY2. Both forms localize to the plasma membrane, but this localization and binding to K-Ras are disrupted by inhibitors of K-Ras membrane anchorage or activity. Mutations at the predicted interface of K-Ras and SPRY2’s C-terminal region affect the interaction. Both full-length SPRY2 and its C-terminal fragment promote the differentiation of C2C12 muscle cells, a process requiring MAPK pathway inhibition. Finally, SPRY2 homo- and hetero-oligomerizes with SPRY4. We propose that active K-Ras recruits SPRY2 dimers to the membrane, where they block Ras effector access.

## Highlights

• Identified eight new modulators of K-Ras4B trafficking and membrane organization
• The proximal hit APLP2 engages with C-Raf and independently and indirectly with K-Ras
• Plasma membrane recruitment of SPRY2 depends on Ras- and SPRY-lipidation
• The C-terminal half of SPRY2 blocks the effector region of Ras

## Graphical abstract

**Figure:**
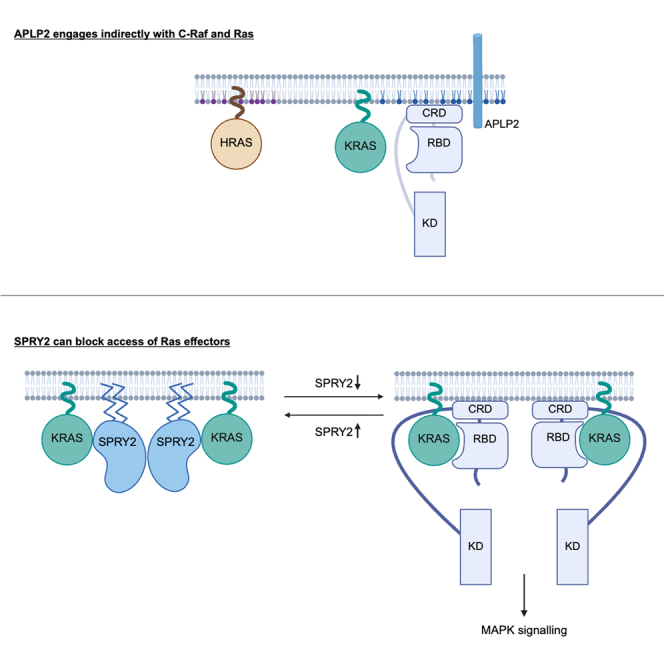

## Introduction

Ras signaling plays a critical role in cell proliferation and differentiation and contributes to a wide range of cellular functions and phenotypes important for health and disease.^1^ The Ras protein operates like a molecular switch inside the plasma membrane, where it links growth factor receptor inputs to effector outputs. Ras effectors bind to Ras in a nucleotide-dependent manner, and their concentration at the plasma membrane leads to functional interactions that influence cell signaling networks.^2^ For instance, Raf family members are canonical Ras effectors that initiate the MAP kinase cascade, which is classically associated with cell proliferation.

The Ras family consists of four isoforms, H-Ras, N-Ras, K-Ras4A, and K-Ras4B (hereafter referred to as K-Ras), that are structurally highly related and share a common set of regulators and effectors.^3^ While their G-domain is 80–90% identical, the C-terminal hypervariable region (HVR) distinguishes them. The HVR of all Ras proteins becomes farnesylated, thus anchoring Ras to cellular membranes. However, isoform-specific functional differences have been observed, and in recent years, this is thought to at least be partly due to isoform-specific differences in the subcellular localization and occupation of a mosaic of different cell surface signaling nanoclusters.^4^^,^^5^

Both K-Ras4B and H-Ras occupy activation state specific nanodomains in the plasma membrane and can modulate each other’s nanoclustering by organizing in particular phosphatidylserine.^6^ Importantly, nanoclusters determine the recruitment efficiency of effectors, which increases signal transmission efficiency across the plasma membrane.^7^^,^^8^ The Ras-isoform specific engagement of Raf effectors is further modulated by domains of the protein that engage the membrane, probably by interacting preferably with certain lipid species.^9^ While nanoclusters may facilitate Raf dimerization, the dimeric state of Raf in turn appears to stabilize Ras nanoclusters.^10^ The best studied nanocluster modulator, galectin-1,^11^ is dimeric at higher concentrations and, by binding to the Ras binding domain of Raf, can increase nanoclustering of active H-Ras specifically.^10^ Recently, a peptide was developed, which interferes with the binding of galectin-1 to the RBD and thus disrupts H-RasG12V nanoclusters.^12^ This example illustrates that any modulator of Ras membrane organization is of high interest as a potential drug target. Furthermore, the mechanistic links between nanoscale spatial organization and Ras isoform functional specificity remain poorly understood because of the challenges in characterizing the protein and lipid compositions of ephemeral signaling domains.

One strategy for understanding the molecular context in which Ras proteins operate has been to employ proteomic analysis of the Ras interactome. Up to 210 proteins were detected as potential direct or indirect Ras interactors capable of being co-immunoprecipitated.^13^ Several groups have since utilized BirA proximity-dependent biotin identification (BioID), able to detect not only direct interactors but also proteins in proximity to Ras.^14^ BirA fused to the Ras N-terminus catalyzes the conjugation of biotin to proteins in ∼10 nm vicinity that can then be identified via streptavidin enrichment and proteomic analysis. Ras proximal proteome studies to date have typically relied on full length wild type and constitutively active Ras mutants.^15^^,^^16^^,^^17^^,^^18^ One of the challenges with this approach is the promiscuity of biotinylation since the proximal proteome of the full cellular itinerary of Ras from initial expression to all its destinations is labeled. Combination with a secondary orthogonal screen has been an effective way for prioritizing hits.^15^ Another refinement has been TurboID, which employs a mutated BirA optimized to significantly reduce the biotinylation timeline from 18 to 24 h to 10 min.^19^

In this study, we aim to identify proteins that regulate K-Ras membrane organization and function in a manner dependent upon its activation state.

## Results

### TurboID identifies the proximal proteome associated with the G-domain and mutationally activated K-Ras

**Figure 1:**
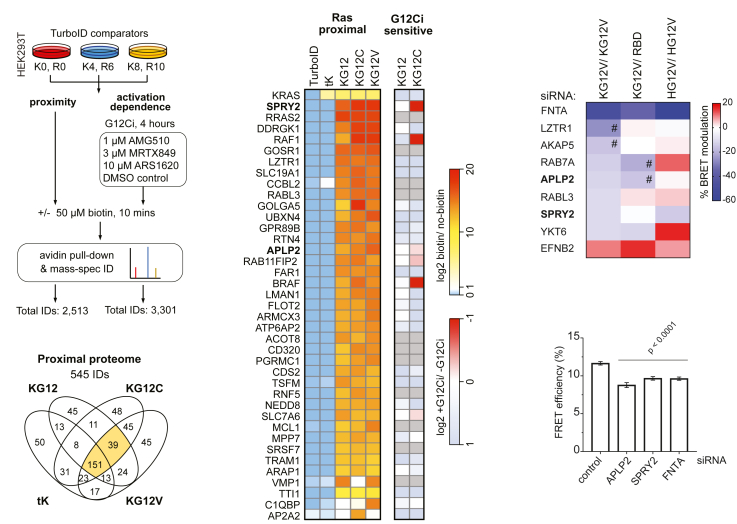
TurboID- and BRET-screening identify bona fide K-Ras G-domain interactors that modulate its membrane organization (A) Schematic of the two TurboID experiments that detected proximity and activation dependent proximity to K-Ras variants. SILAC labeling employs isotopes of arginine (R0, 6, 10) and lysine (K0, 4, 8) to allow mass-spectrometric identification and ratiometric comparison of relative protein biotinylation in each condition following streptavidin enrichment. (B) Venn Diagram by TurboID-construct, wt K-Ras (KG12), K-RasG12D (KG12C), K-RasG12V (KG12V) and tK. Total IDs were all proteins detected in at least one condition across *N* = 3 experiments. The proximal proteome shortlist represents all hits that were ≥ 2-fold enriched versus the equivalent non-biotinylated control condition and also not proximal to the TurboID control. Data are rank order from top to bottom, from most to least enriched across all three variants. (C) Relative enrichment of the 39 proximal hits observed in all three of the full-length K-Ras variant conditions, together with the activation dependence on their proximity. (D) Modulation of the BRET signal after siRNA-mediated knockdown of indicated TurboID hits in BRET-assays to measure K-RasG12V membrane organization (KG12V/KG12V), K-RasG12V/C-Raf-RBD interaction (KG12V/RBD), and H-RasG12V membrane organization (HG12V/HG12V) in HEK cells from *N* = 4 biological repeats. # marks hits with ≥15% BRET modulation. (E) Quantification of FLIM-FRET imaging data of HEK293T cells transfected with mEGFP-K-RasG12V/mCherry-K-RasG12V after siRNA-mediated knockdown of indicated genes of interest. Means ± SEM from *N* = 3 biological repeats are plotted. For APLP2 siRNA from *N* = 2 biological repeats. Per condition, 50–100 cells were analyzed. Statistical comparisons were done using one-way ANOVA and differences to the scrambled siRNA control are indicated. See also Figures S1 and S3.

We employed TurboID to identify proteins that operate in close proximity to K-Ras. Experiments were conducted to first identify the K-Ras proximal proteome and then to identify the dependence of this proximity on the K-Ras activation state (Figure 1A). Stable isotope labeling by amino acids in cell culture (SILAC) was used to allow for ratiometric proteomic comparison of streptavidin enrichment between each set of comparators.^20^ SILAC uses heavy isotope variants of arginine (R) or lysine (K), which allows peptides isolated from samples grown with these isotopes to be distinguished in the mass spectrometer. The available combinations of arginine and lysine isotopes mean that three experimental conditions (triplexes) can be compared in each mass spectrometry run. For proximity analysis, a TurboID + biotin control was included in all comparator triplexes to allow comparison of all conditions, and the other two comparators were each of the K-Ras variants ± biotin. For activation dependence, the comparator triplex consisted of a construct with no treatment, and treatment with biotin with or without K-RasG12C inhibitor (G12Ci) (Figures S1 and S2).

For K-Ras proximity, we compared three full-length K-Ras variants comprising wild type and the G12C and G12V activated mutants of K-Ras that are commonly found in patients with cancer.^21^ We also included the K-Ras fragment tK, which comprises residues 175–188 of the HVR.^22^ While it is targeted to the plasma membrane, it lacks the G-domain (residues 1–165) that is necessary for most, if not all, currently known Ras interactions.^22^ Three biological replicates of each experiment were conducted, and the total number of proteins seen in each condition for the proximal proteome experiment was 545 (Figure 1B and Data S1). Shortlisting was based on observing an average of ≥ 2-fold enrichment over the control without biotin across the replicates, together with also not being proximal to the cytoplasmic TurboID control (≥2-fold + biotin vs. – biotin TurboID). Thus, 190 proteins were identified across all K-Ras variants, with 39 proteins observed just with the full-length variants but not tK, suggesting their proximity depends on the G-domain (Figure 1C).

For activation-dependence of K-Ras proximity, we tested three different selective G12Ci, with K-RasG12C and K-Ras wild type conditions. Direct covalent inhibition of mutant K-Ras is an improvement over previous strategies of comparing wild type with constitutively active K-Ras mutants. The proximity of 132 proteins to K-RasG12C was sensitive to G12Ci (Data S1) and comprised canonical Ras effectors such as Raf and PI3-kinase family members. Raf1 (C-Raf), B-Raf and SPRY2 showed a prominent G12Ci-sensitivity for their K-RasG12C proximity labeling (Figures 1C, S1, and S2). This might be expected for the former two, given that Raf proteins are well-established Ras effectors. Yet for SPRY2, the G12Ci-sensitive proximity suggests an activation state-dependent interaction that was not reported previously.

### Profiling for Ras-membrane organization modulators by bioluminescence resonance energy transfer

To functionally profile proteomic hit proteins impacting on K-Ras membrane organization, we performed bioluminescence resonance energy transfer (BRET) experiments following knockdown of the hit genes using siRNA.

Our primary BRET-screen employed our well-established K-RasG12V-membrane organization BRET-biosensor, consisting of the BRET-pair RLuc8-K-RasG12V and GFP2-K-RasG12V expressed in HEK293-EBNA cells (hereafter HEK). An analogous FRET-biosensor reported on the loss of K-Ras-nanoclustering, membrane anchorage, and -lipidation with a drop in the FRET-signal.^23^ In agreement with this, we previously demonstrated that the K-RasG12V BRET-biosensor responds to an inhibition of Ras trafficking chaperones and Ras lipidation.^24^^,^^25^^,^^26^^,^^27^

Validation of this assay in the screening setting revealed that the ablation of FNTA, the gene encoding for the common ⍺-subunit of farnesyl- and geranylgeranyl-transferases, led to the strongest drop in the BRET signal (Figures S3A and S3B). A clear effect was also observed after knockdown of trafficking chaperones (PDE6D, CALM1), while nanocluster modulators (LGALS3, TP53BP2) had a less pronounced effect (Figure S3B). This latter observation contrasts with our previous FRET measurements, which displayed a much larger dynamic range.^28^^,^^29^^,^^30^^,^^31^

Based on these validation data, we set a 10 % change in the BRET signal as a threshold for high confidence BRET-hits on this primary BRET-screen. A selection of 29 hits from the putative G-domain binder hit list and a meta-analysis of four other K-Ras-proximal proteome studies were then screened (Figure S3C). The meta-analysis identified 1,366 proteins proximal to at least one isoform of Ras.^15^^,^^16^^,^^17^^,^^18^ However, only 12 K-Ras proximal proteins were common to all four other studies and included Raf isoforms and the Ras inactivator NF1 (Data S1). From those proteins that were common to three or four of these studies, we selected a subset of 21 for further investigations.

While 4 of 8 (50%) of the G-domain binder list were confirmed as BRET hits, only 4 of 21 (∼20%) from the meta-analysis list qualified as such, suggesting that our differential TurboID-screening approach is advantageous to identify putative K-RasG12V membrane organization modulators (Figures 1D and S3C). All of the BRET-screen hits reduced K-RasG12V membrane organization BRET after their siRNA-mediated depletion, except for EFNB2, which increased it (Figure 1D).

To functionally profile hits further in secondary BRET screens, we employed two additional biosensors. Using the RLuc8-K-RasG12V and C-Raf-RBD-GFP2 biosensors, we could detect perturbations of Ras effector binding. This included not only direct competition at the effector binding site but also loss of Ras nanoclustering, which we previously showed reduces the recruitment efficiency of effectors from the cytosol as measured by this assay^7^^,^^12^ (Figures 1D and S3D). The third BRET-assay served to assess relative K-Ras selectivity by detecting H-Ras membrane organization using RLuc8-H-RasG12V and GFP2-H-RasG12V as biosensors (Figures 1D and S3D).

Based on this characterization, we decided to focus on two genes from the BRET-screen hits, APLP2 and SPRY2. APLP2 is not known for any function in concert with K-Ras. SPRY2 belongs to the family of four SPRY- or sprouty-proteins, which are frequently observed hits in Ras proximal proteome screens.^15^^,^^16^^,^^17^^,^^18^ To confirm our BRET-screen results for these proteins, we used the more sensitive fluorescence lifetime imaging microscopy (FLIM)- Förster resonance energy transfer (FRET) approach. FLIM-FRET data supported a highly significant reduction in K-RasG12V membrane-FRET upon knockdown of either APLP2 or SPRY2 (Figures 1E, S3E, and S3F). Given that it is unknown how these proteins bind to Ras and impact its membrane organization, we continued their investigation.

### APLP2 engages indirectly with C-Raf and less with oncogenic K-Ras

Amyloid precursor-like protein 2 (APLP2) is a member of the evolutionary conserved amyloid-precursor-like (APP) protein family. APLP2 is a ubiquitously expressed *trans*-membrane glycoprotein that becomes proteolytically activated by secretases.^32^ APLP2 is aberrantly expressed in different types of cancers, with overexpression as well as proteolytic cleavage positively correlating with cell growth and migration.^32^ In the genetically engineered KRAS-G12D-driven KPC-mouse model for spontaneous pancreatic cancer, deletion of APLP2 significantly prolonged survival and reduced metastasis.^33^ Given this genetic interaction of K-Ras and APLP2, we wanted to validate the biochemical interaction of these two proteins.

**Figure 2:**
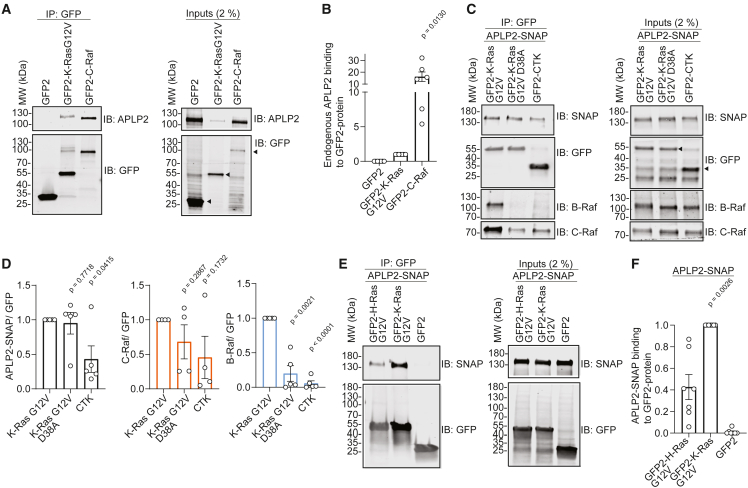
APLP2 preferentially engages with C-Raf and less with K-RasG12V (A and B) Representative blots of GFP-Trap pull-downs (left) of endogenous APLP2 from HEK lysates (right) using GFP2-K-RasG12V or GFP2-C-Raf (A) with quantified means ± SEM from *N* = 6 or 7 biological repeats of pull-down data analyzed using Welch's *t* test (B). Arrowheads mark specific bands of expressed constructs. (C and D) Representative blots of GFP-Trap pull-downs (left) of APLP2-SNAP and endogenous C-Raf and B-Raf in the presence of the former from HEK lysates (right) using GFP2-K-Ras derived constructs as indicated with GFP2-CTK serving as control (C) with quantified means ± SEM from *N* ≥ 4 biological repeats of pull-down data analyzed using Welch's *t* test (D). Arrowheads mark specific bands of expressed constructs. (E and F) Representative blots of GFP-Trap pull-downs (left) of APLP2-SNAP from HEK lysates (right) using GFP2-K-RasG12V, GFP2-H-RasG12V, or GFP2 (control) as indicated (E) with quantified means ± SEM from *N* = 7 biological repeats of pull-down data analyzed using Welch's *t* test (F).

Using co-immunoprecipitation experiments, we characterized the engagement of APLP2 with Ras and Raf proteins. Endogenous APLP2 was ∼15-fold more co-immunoprecipitated with GFP2-tagged C-Raf than with GFP2-tagged K-RasG12V (Figures 2A and 2B). This was in line with the BRET-profiling data, showing that the depletion of APLP2 affects both the K-RasG12V/K-RasG12V- and K-RasG12V/C-Raf-RBD-BRET signal (Figure 1D).

To understand if the K-Ras interaction was sensitive to the D38A-mutation, which abrogates interactions with all major effectors,^34^ we performed GFP-Trap pull-down experiments of C-terminally SNAP-tagged full length APLP2 with GFP2-K-Ras constructs from HEK cell lysates. All signal obtained with GFP2-CTK, which encodes only the membrane anchoring HVR sequence (residues 166–188) of K-Ras, was considered background. While both endogenous C-Raf and B-Raf co-immunoprecipitated more with K-RasG12V than K-RasG12V-D38A, APLP2-SNAP co-immunoprecipitated to about the same extent with both K-Ras mutants (Figures 2C and 2D). This insensitivity of APLP2 to the D38A mutation suggests an interaction with a pool of C-Raf that is not bound to K-Ras but still in its proximity.

BRET-screening data suggested a specific effect of APLP2 on K-RasG12V- but not on H-RasG12V-membrane organization (Figure 1D). In line with this, GFP2-K-RasG12V co-immunoprecipitated > 2-fold more APLP2-SNAP than GFP2-H-RasG12V (Figures 2E and 2F).

In conclusion, both BRET-validation and co-immunoprecipitation data suggest that APLP2 may only indirectly engage with C-Raf and less so with K-RasG12V, while interaction with H-RasG12V is even lower. One possibility to explain these data is via the modulation of the membrane environment by APLP2, which may thus promote Ras/Raf-complex formation at the plasma membrane and (Raf-dependent) K-Ras nanoclustering, consistent with our previous model for active nanoclusters.^10^^,^^12^^,^^29^ However, the data provided here merely validate APLP2 engagement with C-Raf, K-RasG12V, and H-RasG12V, and the proposed explanation is purely speculative, pending further investigations.

### SPRY2 interacts with oncogenic K-Ras predominantly through its C-terminal region

SPRY2 is ubiquitously expressed and the evolutionarily most conserved member of the four human or mouse SPRY proteins.^35^^,^^36^ SPRY proteins are characterized by their C-terminal cysteine-rich domain (CRD), which contains several palmitoylation sites that are important for their localization to the plasma membrane.^37^^,^^38^ The N-terminus of SPRYs is more diverse and features a c-Cbl-tyrosine kinase-binding (Cbl-TKB) motif followed by a serine-rich motif (SRM). At the plasma membrane, SPRY2 is phosphorylated on a conserved Tyr55 in the Cbl-TKB motif, which is associated with the ubiquitin mediated regulation of SPRY stability.^36^

Drosophila SPRY was identified as an antagonist of the Ras-MAPK pathway during fly trachea development.^39^ This ability to down-regulate Ras signaling in response to a variety of growth factor stimuli is preserved in vertebrates.^40^ It was previously proposed that phosphorylated SPRY negatively regulates Ras-MAPK-signaling by sequestering the adaptor protein Grb2, thus preventing the activation of SOS.^40^ Others suggested SPRY proteins inhibit the MAPK- but not the PI3K-pathway downstream of Grb2-SOS.^41^ However, several biochemical and proteomics studies have identified SPRY2 as a potential K-Ras-^15^^,^^16^^,^^42^ or H-Ras interactor.^43^

**Figure 3:**
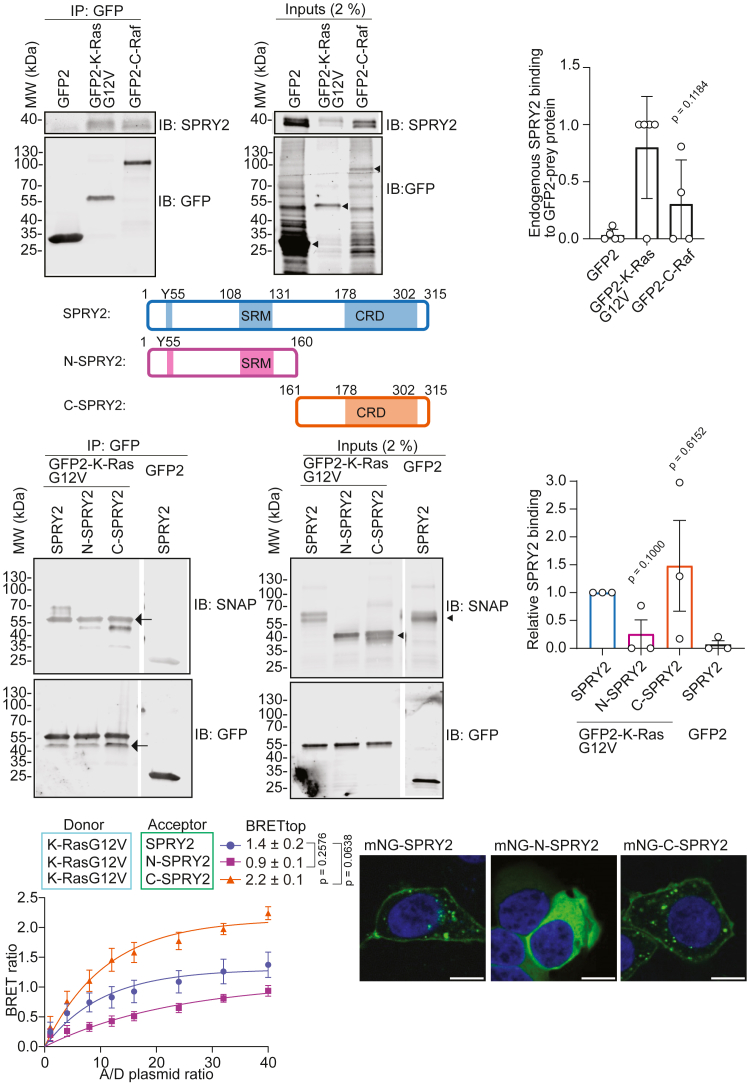
SPRY2 interacts with K-Ras predominantly via its C-terminal half (A and B) Representative blots of GFP-Trap pull-downs (left) of endogenous SPRY2 from HEK lysates (right) using GFP2-K-RasG12V or GFP2-C-Raf (A) with quantified means ± SEM from *N* = 5 biological repeats of pull-down data analyzed using Welch's *t* test (B). Arrowheads mark specific bands of expressed constructs. (C) Schematic of the SPRY2-fragment constructs used in pull-down and BRET experiments. SRM indicates serine rich motif, and CRD cysteine rich domain. The Y55 marks the tyrosine of the Cbl-TKB binding motif. (D and E) Representative blots of GFP-Trap pull-downs (left) using indicated SNAP-tagged SPRY2-constructs from lysates of HEK (right) expressing GFP2-K-RasG12V or GFP2 (control) (D) with quantified means ± SEM from *N* = 3 biological repeats of pull-down data analyzed using Welch's *t* test (E). Arrowheads mark specific bands of expressed constructs, while the arrows indicate a non-specific band. (F) BRET-titration curves of the nL-K-RasG12V interaction with mNG-tagged SPRY2 fragments as introduced in (C) acquired in HEK cells from *N* = 3 biological repeats. Means ± SEM of BRETtop were analyzed using One-Way Brown-Forsythe and Welch ANOVA tests with Dunnett's T3 correction for multiple comparisons. (G) Confocal imaging shows the subcellular localization of mNG-tagged SPRY2-constructs in HEK cells. Scale bars = 10 μm See also Figure S4.

We therefore wanted to identify the structural determinants for the interaction between K-Ras and SPRY2. Using co-immunoprecipitation and cellular BRET-interaction studies, we verified our BRET-screening data, which suggested an interaction preference for K-Ras but no impact of SPRY2-depletion on effector engagement (Figure 1D). Indeed, GFP-Trap pull-down confirmed that endogenous SPRY2 from HEK cell lysates binds ∼2.5-fold more to GFP2-K-RasG12V than GFP2-C-Raf (Figures 3A and 3B).

To identify which part of SPRY2 interacts with K-RasG12V, we studied the interactions of SNAP-tagged N-terminal SRM-comprising (N-SPRY, residues 1–160) and C-terminal CRD-comprising fragments (C-SPRY2, residues 161–315) (Figure 3C). As compared to the full-length protein, N-SPRY2 showed a ∼5-fold reduced interaction with K-RasG12V, while the C-SPRY2 interaction was ∼1.5-fold increased (Figures 3D and 3E). This was confirmed by BRET-interaction data between mNeonGreen (mNG)-tagged SPRY2-fragments and nanoLuc (nL)-tagged K-RasG12V. The BRET signal was 1.6-fold reduced with N-SPRY2, while that of C-SPRY2 was 1.6-fold increased (Figure 3F). The same order of BRET-interactions was seen with H-RasG12V (Figure S4A), which engaged, however, ∼25% less than K-RasG12V with SPRY2 based on co-immunoprecipitation data (Figures S4B and S4C). MAPK-signaling suppression by SPRY2-fragments under the tested conditions were moderate but followed the order of Ras engagement (Figures S4D and S4E).

High BRET levels can emerge from co-localization at the plasma membrane. We therefore analyzed the distribution of the mNG-tagged SPRY2-constructs in HEK cells by confocal imaging. When observed under normal serum conditions, full length SPRY2 was found at the plasma membrane both in the presence (Figure S4F) and absence of co-expressed mCherry-K-RasG12V (Figure 3G). Also, C-SPRY2 was localized in this way, while N-SPRY2 was predominantly in the cytoplasm (Figure 3G). This distribution pattern of the constructs can be explained by the fact that the C-terminal CRD of SPRY2 contains 26 cysteine residues, some of which are known to be palmitoylated. Palmitoylation is important for targeting SPRY2 to the plasma membrane, and mutation of two cysteine residues, C265 and C268, re-distributes SPRY2 to the cytoplasm.^38^

Thus, the C-terminal half of SPRY2 is sufficient to translocate it to the plasma membrane, where it can engage with RasG12V.

### SPRY2 recruitment to the plasma membrane is sensitive to lipidation and K-RasG12C inhibitors

We next wanted to understand if SPRY2 plasma membrane localization depends on prenylated and active K-Ras. We therefore first treated cells with mevastatin, which blocks the synthesis of prenyl-pyrophosphate in the mevalonate pathway and thus Ras prenylation and membrane organization associated with BRET.^24^ Given that the C-terminal palmitoylation of SPRY2 is required for its membrane anchorage, we reasoned that 2-bromopalmitate (2-BP) would directly block SPRY2 plasma membrane binding.^38^^,^^44^

We first confirmed that the nanoclustering-associated BRET of farnesylated K-RasG12V was significantly abrogated by mevastatin and that of palmitoylated and farnesylated H-RasG12V was sensitive to both mevastatin and/or 2-BP (Figures S5A and S5B).

**Figure 4:**
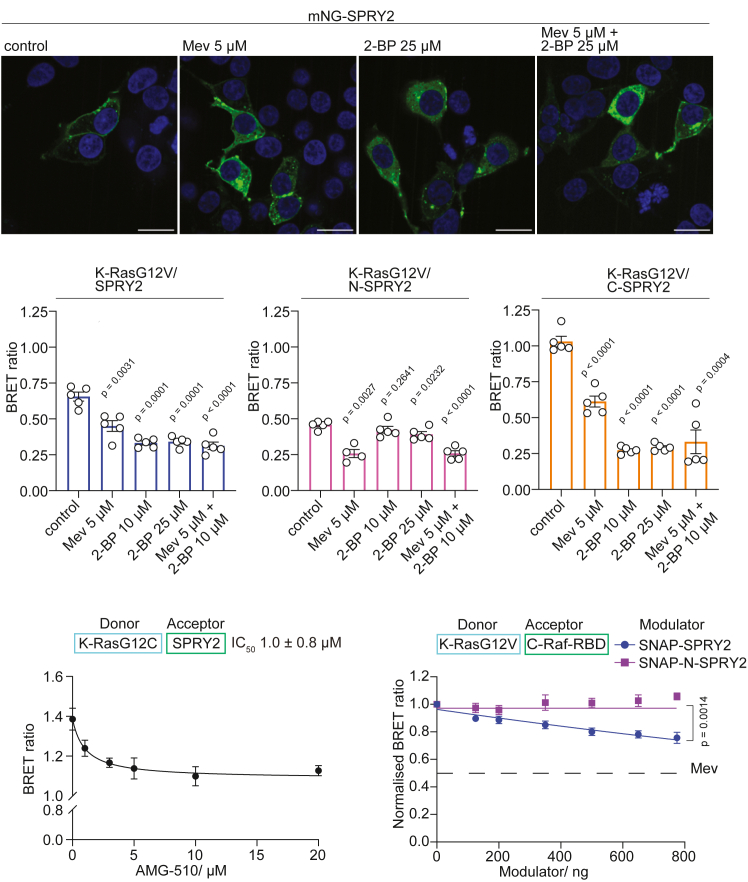
K-RasG12V engagement with SPRY2 is sensitive to lipidation inhibitors (A) Confocal imaging shows that the plasma membrane localization of mNG-tagged SPRY2 in HEK cells is sensitive to lipidation inhibitors mevastatin (Mev) and/or 2-bromopalmitate (2-BP). Scale bars = 20 μm (B–D) Effect of mevastatin (Mev) and/or 2-bromopalmitate (2-BP) on interaction-BRET of nL-K-RasG12V with mNG-SPRY2 (B), mNG-N-SPRY2 (C) or mNG-C-SPRY2 (D) in HEK cells (donor:acceptor plasmid ratio = 1:8). Plotted are means ± SEM from *N* = 5 independent biological repeats analyzed using Welch's *t* test (B–D). (E) Dose-dependent disruption of nL-K-RasG12C/mNG-SPRY2 BRET-interaction after treatment with G12Ci AMG510 in HEK cells (donor:acceptor plasmid ratio = 1:40). Plotted are means ± SEM from *N* = 3–9 independent biological repeats. (F) Dose-dependent effect of SNAP-tagged SPRY2 or N-SPRY2 expression on nL-K-RasG12V/mNG-C-Raf-RBD BRET in HEK cells (donor:acceptor plasmid ratio = 1:8). Plotted are means ± SEM from *N* = 4 independent biological repeats with values at highest modulator concentration compared using Welch's *t* test. Dashed line indicates the response to 5 μM mevastatin (Mev) treatment, indicating full plasma membrane displacement. See also Figure S5.

Confocal imaging data suggested that mevastatin treatment led to the emergence of a cytoplasmic pool of SPRY2, similar to that observed after treatment with 2-BP. Combined treatment with mevastatin and 2-BP most efficiently redistributed SPRY2 to the cytoplasm (Figure 4A). We then used BRET between K-RasG12V and SPRY2-fragments to quantify the impact of the lipidation inhibitors on their co-localization-dependent interaction. If both proteins interact at the membrane, high BRET is expected, also due to the up-concentration at the membrane.^23^ However, if one or both cannot localize to the membrane due to blocked lipidation the BRET signal would be lowered. BRET of full length mNG-SPRY2 with nL-K-RasG12V was significantly reduced after mevastatin treatment, but even more so after 2-BP incubation or treatment with both lipidation inhibitors (Figure 4B). While the N-SPRY2 fragment localized predominantly to the cytoplasm (Figure 3G), its BRET with K-RasG12V was still sensitive to mevastatin, suggesting that membrane localized K-Ras can still engage with N-SPRY2. Consistent with N-SPRY2 being devoid of the C-terminal palmitoylation sites, 2-BP did not affect the BRET, while the combination of mevastatin and 2-BP reduced the BRET to the same extent as mevastatin alone (Figure 4C). C-SPRY2 showed the highest BRET with K-RasG12V, which was significantly reduced after mevastatin treatment and even more after 2-BP treatment, while the combination of both inhibitors could not further reduce the BRET (Figure 4D).

Our TurboID analysis indicated that SPRY2 interacts with K-RasG12C in a G12Ci-sensitive manner, just as well-established effectors C-Raf (Raf1) and B-Raf (Figures 1C and S1). We therefore next verified this again using BRET. In agreement with the TurboID data, the maximal interaction-BRET signal of nL-K-RasG12C and mNG-SPRY2 was dose-dependently reduced (IC_50_ = 1 ± 0.8 μM) after treatment with G12Ci AMG510/sotorasib (Figure 4E).

The K-RasG12V effector binding interface dependent binding of SPRY2 was further supported by BRET experiments, where we titrated SNAP-tagged SPRY2 or N-SPRY2 against the nL-K-RasG12V/mNG-C-Raf-RBD BRET-pair (Figure 4F). While increasing concentrations of N-SPRY2 did not alter the BRET, full length SPRY2 led to a ∼50% reduction at the highest tested expression level relative to the mevastatin treatment control level (Figures 4F, S5C). Conversely, mCherry-tagged C-Raf could dose-dependently reduce BRET between nL-K-RasG12V/mNG-SPRY2 (Figures S5D and S5E), supporting that both SPRY2 and effector compete for the same binding site on K-RasG12V.

These data suggest that SPRY2 is recruited to farnesylated, active K-Ras at the plasma membrane via its C-terminal domain, which anchors it there via its palmitoyl moieties.

### SPRY2 binding to K-RasG12V is modulated by predicted interface mutations

**Figure 5:**
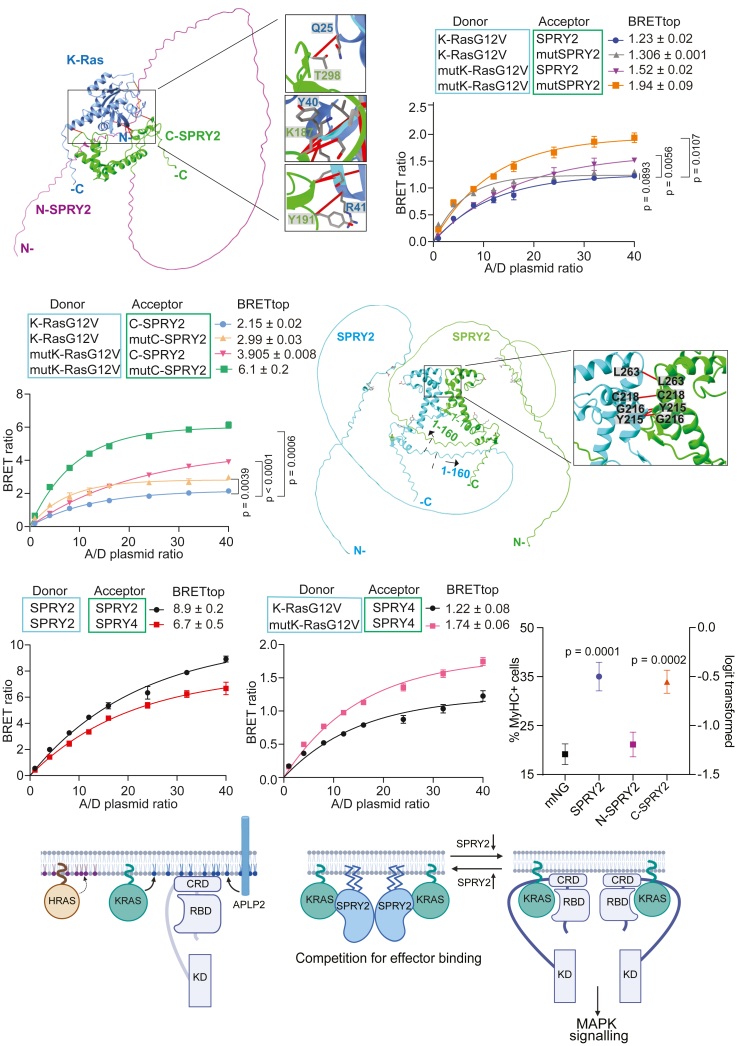
SPRY2 is recruited to active K-Ras on the plasma membrane (A) AlphaFold 3 prediction of K-Ras/SPRY2 complex, with zoom-in showing residues with putative side chain interactions. (B and C) BRET-titration curves of the nL-tagged K-RasG12V or K-RasG12V-Q25A/Y40A/R41A (mutK-RasG12V) interactions with mNG-tagged SPRY2 or SPRY2-K187A/Y191A/T298A (mutSPRY2) (B), respectively corresponding C-SPRY2 variants (C) in HEK cells from *N* = 2-4 independent biological repeats. Means ± SEM of BRETtop were analyzed using One-Way Brown-Forsythe and Welch ANOVA tests with Dunnett's T3 correction for multiple comparisons. (D) AlphaFold 3 prediction of the SPRY2/SPRY2 interaction, with zoom-in showing residues within 3 Å distance. (E) BRET-titration curves of the nL-SPRY2/mNG-SPRY2 and nL-SPRY2/mNG-SPRY4 interaction in HEK cells with means ± SEM of BRETtop from *N* = 4 independent biological repeats. (F) BRET-titration curve of the wt or mutant nL-K-RasG12V with mNG-SPRY4 in HEK cells with means ± SEM of BRETtop from *N* = 3–6 independent biological repeats. (G) Flow cytometric quantification of MyHC terminal differentiation marker expression in C2C12 cells in low serum for 72 h after transfection with mNG-SPRY2, mNG-N-SPRY2, or mNG-C-SPRY2 constructs. Means ± SD are plotted from *N* = 3 biological repeats. Statistical analysis was done using one-way ANOVA and Dunn’s post hoc test. (H) Schematic illustrating our speculative models for how APLP2 (left) and SPRY2 (right) impact Ras membrane organization and activity. See the main text for more details. See also Figures S6 and S7.

To identify the interface that mediates the interaction between K-Ras and SPRY2, we predicted the structure of the complex using AlphaFold 3 (AF3).^45^ The predicted K-Ras/SPRY2 complex suggested binding of N-terminal but more so of C-terminal fragment residues of SPRY2 to the effector binding region of K-Ras (Figure 5A). Major putative side-chain contacts between C-terminal SPRY2 residues and K-Ras residues were T298-Q25, K187-Y40, and Y191-R41, respectively (Figure 5A). While these residues were phylogenetically conserved in mouse, chick, and mostly also in frog, both on the SPRY2 and K-Ras sides (Figures S6A–S6C), conservation among other SPRY-family members was limited (Figure S6D), suggesting a specialized role of SPRY2 in this context.

We mutated the six potentially contacting residues in both proteins to alanines and monitored how interaction-BRET between them was altered. Surprisingly, the interaction of K-RasG12V- Q25A/Y40A/R41A (mutK-RasG12V) with SPRY2-K187A/Y191A/T298A (mutSPRY2) showed a significantly increased BRET (Figure 5B). Both mutSPRY2 and mutC-SPRY2 localized as the parent (Figure S7A). As expected by the location of the mutations near the effector binding sequence stretch of K-Ras (residues 28–39), BRET of mutK-RasG12V and the C-Raf-RBD showed a significant loss (Figure S7B). Also, mixed BRET-pairs of K-RasG12V and mutSPRY2 or mutK-RasG12V and wt SPRY2 showed an elevated BRET signal (Figure 5B). These differences were even more pronounced when examining these mutations only in the context of C-SPRY2 (Figure 5C). These observations were somewhat explained when examining the AF3-prediction of the mutant complex, which showed an increase in the number of < 3 Å contacts to 25, as compared to 13 in the wt complex (Figures S7C and S7D).

The paralog most related to SPRY2 is SPRY1, followed by SPRY4 and SPRY3; however, the expression of the latter is restricted to the brain.^35^ Several other studies of the proximal proteome identified in particular SPRY2 and SPRY4, but not SPRY3, as potential interactors, notably of activated K-Ras as compared to the other Ras isoforms.^15^^,^^16^^,^^17^^,^^42^^,^^46^ Additionally, SPRY-proteins were suggested to homo- and hetero-di/oligomerize via their CRD.^47^ Consistently, AF3-predicted dimers of SPRY2 with a dimer interface that was compatible with concurrent K-Ras binding (Figure 5D). This was supported by BRET-experiments showing a high BRETtop value for the SPRY2/SPRY2 BRET-pair (Figure 5E). Likewise, BRETtop for the heterodimer of SPRY2/SPRY4 was high, supporting that SPRY-proteins can hetero-di/oligomerize. In line with an engagement of SPRY4 by active K-RasG12V, the BRET signal between the two was as high as that with SPRY2 (Figure 5F) and likewise increased by mutations Q25A/Y40A/R41A in K-RasG12V (Figure 5F).

SPRY-proteins have been implicated in various biological contexts as suppressors of Ras-MAPK-signaling. In cancer cells, their role is contentious, with some reporting tumor suppressive activities, while others consider it oncogenic.^48^ Current evidence suggests that the normal biological function of SPRY-proteins is associated with stem cell quiescence, such as those of muscle cells.^49^ However, in the murine C2C12 skeletal muscle cell line, SPRY2 expression suppresses ERK activation and promotes differentiation.^50^ This is in line with the suppression of MAPK signaling that needs to occur to initiate muscle cell differentiation.^51^

We therefore used our well established C2C12 muscle cell differentiation assay to assess the functional effect of mNG-tagged SPRY2 fragment expression.^52^^,^^53^ Differentiation is quantified by the flow-cytometric measurement of the late differentiation marker myosin heavy chain (MyHC). As expected, the expression of full length SPRY2 significantly increased differentiation. Consistent with C-SPRY2 binding to and blocking active K-Ras at the plasma membrane (Figure 4) and suppressing MAPK-signaling the strongest (Figures S4D and S4E), this fragment was sufficient to significantly increase differentiation (Figure 5G). By contrast, N-SPRY2, which alone only poorly engages with K-RasG12V (Figure 3F), had no effect on differentiation (Figure 5G).

Based on our data, we propose a speculative model wherein a dimer of SPRY2 with other SPRY proteins is recruited to two active K-Ras proteins by directly binding to their effector binding regions (Figure 5H). We further speculate that membrane contacts of the SPRY2/K-Ras complex localize it in lipid-nanodomains that are also employed by Raf proteins. Thus, SPRY proteins block access to Raf and downregulate specifically Ras-MAPK signaling, which in the muscle C2C12 cell line supports differentiation.

## Discussion

Using proteomics- and BRET screens, we have identified and categorized two proteins of the proximal K-Ras proteome, which modulate its membrane organization. Proximity proteomics approaches are inherently noisy. We identified 190 proteins proximal to full length K-Ras proteins. 70% of these proteins have been identified in at least one previous K-Ras BioID study. However, only six (ARAF, BRAF, CRAF, YKT6, MPP7, and EPHA2) of our proteomics hits were classified as proximal to K-Ras in all previous studies (Data S1). Comparison of full-length K-Ras with the minimal K-Ras HVR fragment, tK, that is unable to engage with effectors, together with the determination of the sensitivity of the K-RasG12C proximal proteome to G12Ci, variously allowed the prioritization of hits for further study. Secondary screening using BRET allowed further stratification of hits. We noticed that the dynamic range in the FLIM-FRET-based hit validation experiments is far greater than in the BRET-screen. However, plate-reader based BRET screens are easier to implement than microscopy based FLIM-FRET screens.^54^

We focused our more detailed validation on two hits, APLP2, which is an indirect modulator of K-Ras membrane organization and SPRY2, which appears to be a direct, effector-like interaction partner of K-Ras. By impacting on its local lipid environment, APLP2 may pre-organize or corral a pool of C-Raf not binding to K-Ras, but in its nanoscale vicinity. This would be enabled through changes in the lipid environment or via up-concentration of C-Raf to promote Raf-dimers and thus Raf-dependent nanoclustering of K-Ras^10^^,^^12^ (Figure 5H). This speculative model is merely based on our hit validation experiments, and more extensive investigations are required to prove it. For instance, to test for the Ras-dependent enrichment of C-Raf at the membrane that would subsequently be corralled by APLP2, one could examine the C-Raf-R89L mutant. The R89L mutation abrogates binding to Ras and should therefore reduce the pool of membrane bound C-Raf that could be engaged by APLP2. Moreover, the identification of the protein domains that mediate the engagement may help to clarify the mechanism. Given the unclear relation between APLP2 and K-Ras in cancer or other diseases, it may be interesting to follow up on their shared biology.

SPRY-proteins have been implicated as negative regulators specifically of MAPK-signaling.^36^ While they are dysregulated in cancer,^48^ their normal biological role appears to be associated with the quiescence of stem cells, where they suppress MAPK activity.^49^^,^^55^^,^^56^^,^^57^ For instance, re-entry of muscle stem cells into the quiescent state depends on SPRY1 expression.^49^ The importance of this process is underscored by the fact that the methylation of the SPRY1 gene is observed in muscle cells with aging, which would disrupt re-quiescence of stem cells and consequently foster their demise.^58^

The C2C12 cell line contains only ∼2% muscle stem cells; hence, the quiescent state is less relevant in our assay, where we instead modulate the fate of the committed myoblasts that make up the majority of cells in this cell line.^53^ This is supported by data showing that the ablation of SPRY1 decreases the expression of the muscle differentiation marker MyHC in C2C12 cells.^59^ Our data are therefore consistent with the SPRY2-mediated downregulation of MAPK-signaling to facilitate cell differentiation.^51^ Understanding this native biological context has implications, e.g., in cancer, where SPRY proteins are typically downregulated.^60^ This may suggest that loss of SPRY perturbs the quiescence of stem-like cells, potentially also cancer stem cells.^61^ In addition, proper terminal differentiation may be compromised. Our mechanistic investigations further suggest that SPRY2 resides in an autoinhibited state in the cytoplasm, which is relieved by the binding of its C-terminal half to active Ras at the plasma membrane. While the N-SPRY alone shows only very weak or no binding to K-RasG12V on its own (Figures 3D–3F), it may support Ras engagement in the full-length context as suggested by the AlphaFold3 model (Figures 5A, S7C, and S7D).

In line with previous reports, plasma membrane anchorage of SPRY is sensitive to the inhibition of palmitoylation, consistent with several C-terminal cysteines that can become palmitoylated.^37^^,^^38^ We propose that residues of this C-terminal part predominate in making contacts with the effector binding region of K-Ras; however, N-terminal residues become involved. In support of our own observations that revealed only a modest effect under the tested conditions (Figures S4D and S4E), a suppression of MAPK-activity by the C-terminal residues 176–315 of SPRY2, but not the N-terminal residues 2–172, has been reported previously.^62^ The modest effect in our data may be explained by the fact that serum starvation would also decrease Ras-GTP levels and thus SPRY2 recruitment.

The association of SPRY2 with C-Raf seems less prominent but agrees with published results.^63^ This interaction may occur in different complexes outside of the plasma membrane. Furthermore, we provide evidence that SPRY2 can homo-di-/oligomerize and hetero-di-/oligomerize with SPRY4, consistent with previous reports of their interaction.^63^^,^^64^ Given that phylogenetically conserved, predicted interface residues K187, Y191, and T298 of SPRY2 are only moderately conserved in other SPRY-proteins, we propose that ubiquitously expressed SPRY2 serves as the main recruiter of various combinations of SPRY2 homo- and heterodimers. In our proposed model, homo- and hetero-di-/oligomers of SPRY2 bind to the effector binding region of active Ras, which blocks access to effectors that prefer the same lipid domains. Thus, Ras activity is essentially clamped down by SPRY-proteins. In this context, we suggest that an amphipathic helix at the C-terminus of SPRY2 is positioned to insert into the membrane and mediates the lipid selectivity. The complex of dimeric Ras that is scaffolded on SPRY-dimers would explain the observed BRET-signature that was indicative of SPRY-dimers stabilizing dimeric Ras nanocluster. Currently, only the structure of short phosphorylated SPRY2 and SPRY4 peptides centered around Y55 bound to a c-Cbl TKB domain exists.^65^^,^^66^ Our study underscores the need for comprehensive structural studies of SPRY/Ras complexes, such as computational docking studies, small-angle X-ray scattering, or NMR analysis on nanodiscs, to examine our structural predictions experimentally and for studies elaborating the exact mechanism and biological context of SPRY-mediated Ras-MAPK-pathway suppression. A more thorough understanding of both the APLP2 and SPRY biology is furthermore warranted before they can be nominated as drug targets.

### Limitations of the study

In this study, we reported the identification of putative interaction partners of K-Ras that act on its membrane organization. The list of eight hits shortlisted after BRET-assay validation would need further in-depth characterization of each hit in biochemical and cell biological studies to clearly establish them as interactors and understand their mechanism of action.

For the suggested interaction between APLP2 with C-Raf and K-RasG12V, we tentatively propose an indirect engagement that is mediated via the membrane. More extensive studies are needed to clearly prove such a mechanism, which is currently only based on our validation data. It remains very challenging to disentangle the binding contributions of membrane-associated proteins. To continue the characterization of the single-span transmembrane protein APLP2, one would next aim at identifying the protein domain that mediates the C-Raf engagement. The intracellular part of the protein is most likely to contain such a candidate domain or region. However, given that the lipid environment may be perturbed, the transmembrane and extracellular parts could be relevant as well. Biophysical studies using FRET and BRET, as well as superresolution microscopy methods and single molecule tracking, could help to investigate this potentially non-classical, membrane mediated engagement in the native cellular membrane context.^67^^,^^68^^,^^69^

Further, we have not provided unequivocal proof of the direct interaction of SPRY2 and Ras proteins. This would require purification of interacting protein fragments with subsequent biochemical characterization, such as by using co-immunoprecipitation, surface plasmon resonance spectroscopy, structural studies, and related approaches. The impact of SPRY2 on Ras biochemical properties, such as nucleotide exchange, could also be a subject of further investigations.^70^

Regarding the biological function of SPRY2, we employed the C2C12 cell differentiation assay, where its ability to suppress MAPK-signaling facilitates skeletal muscle cell differentiation. However, as we also explain, this may not be the main native function of SPRY2 in this context. Our work encourages additional cell biological studies will be required to understand the native context where SPRY proteins suppress Ras-MAPK signaling.

## Resource availability

### Lead contact

Further information and requests for resources and reagents should be directed to and will be fulfilled by the lead contact, Prof. Dr. Daniel Kwaku Abankwa (daniel.abankwa@uni.lu).

### Materials availability

Plasmids generated in this study will be available from the lead contact upon request.

### Data and code availability

• The mass spectrometry proteomics data have been deposited to the ProteomeXchange Consortium via the PRIDE partner repository with the dataset identifier PRIDE: PXD067415.
• All other data reported in this study or any additional information for reanalysis can be shared upon request by the lead contact. This study does not report new code.

## Acknowledgments

LC-MS samples were run by Warwick Scientific Services (University of Warwick, UK) and the Centre for Proteome Research (University of Liverpool, UK). We thank Dr. Cleidi Zampronio and Dr. Philip Brownridge for their assistance with these experiments. This work was supported by grants from the 10.13039/501100000268BBSRC (BB/T012757/1) to IAP and the Luxembourg National Research Fund (10.13039/501100001866FNR) AFR/17927850/Duval C./Kruptor to CJD and INTER/10.13039/100014013UKRI/19/14174764 to D.K.A.

## Author contributions

D.K.A and I.A.P. conceived the design and supervised the work. D.K.A., K.P., I.A.P., and F.E.H. critically revised the article. K.P., F.I.H., C.J.D., G.M., C.L., F.A.S., S.P.L.M., I.A.P., and D.K.A. contributed to the acquisition, analysis, or interpretation of data for the work. D.K.A., K.P., I.A.P., and F.E.H. drafted the article. All gave final approval and agreed to be accountable for all aspects of work, ensuring integrity and accuracy.

## Declaration of interests

The authors declare no competing interests.

## STAR★Methods

### Key resources table

**Table:** 

REAGENT or RESOURCE	SOURCE	IDENTIFIER
**Antibodies**		

Mouse monoclonal anti-GFP (GF28R)	Invitrogen	MA5-15256; RRID: AB_1097928
Mouse monoclonal anti-HA.11 (16B12)	BioLegend	901502; RRID: AB_2565007
Mouse monoclonal anti-Beta Actin antibody (2D4H5)	Proteintech	66009-1-Ig; RRID: AB_2687938
Mouse monoclonal anti-Beta Actin antibody (AC-15)	Sigma	A5441; RRID: AB_476744
Mouse monoclonal anti-GAPDH (GAPDH71.1)	Sigma	G8795; RRID: AB_1078991
Rabbit polyclonal anti-SNAP	New England Biolabs	P9310S; RRID: AB_10631145
Mouse monoclonal anti-B-Raf (F-7)	Santa Cruz Biotechnology	sc-5284; RRID: AB_626760
Rabbit polyclonal anti-C-Raf	Cell Signaling Technology	9422; RRID: AB_390808
Rabbit monoclonal anti-phospho-C-Raf (Ser338) (56A6)	Cell Signaling Technology	9427; RRID: AB_2067317
Rabbit monoclonal anti-p44/42 MAPK (Erk1/2) (137F5)	Cell Signaling Technology	4695; RRID: AB_2067317
Rabbit polyclonal anti- p44/42 MAPK (Erk1/2)	Cell Signaling Technology	9102; RRID: AB_330744
Rabbit monoclonal anti-phospho-p44/42 MAPK (Erk1/2) (Thr202/Tyr204) (D13.14.4E)	Cell Signaling Technology	4370; RRID: AB_2315112
Mouse monoclonal anti-phospho-p44/42 MAPK (pErk1/2) (E10)	Cell Signaling Technology	9106; RRID: AB_331768
Mouse monoclonal anti-human K-Ras (2C1)	Lifespan Bio	LS-C175665; RRID: AB_3713472
Rabbit monoclonal anti-pan-Ras antibody (EP1125Y)	Abcam	ab52939; RRID: AB_2121042
Rabbit monoclonal anti-Spry2 (D3G1A)	Cell Signaling Technology	14954; RRID: AB_2798658
Rabbit polyclonal anti-APLP2	Abcam	ab137597
Rabbit polyclonal anti-APLP2	Proteintech	1504-1-AP; RRID: AB_228959
IRDye 800CW Streptavidin	Li-Cor Biosciences	926–32230
IRDye 680RD Donkey anti-Mouse IgG Secondary Antibody	Li-Cor Biosciences	926–68072; RRID: AB_1095362
IRDye 800CW Goat anti-Rabbit IgG Secondary Antibody	Li-Cor Biosciences	926–32211; RRID: AB_621843
eFluor 660-conjugated anti-myosin 4 (myosin heavy chain, MyHC) antibody	ThermoFisher Scientific	50-6503-82; RRID: AB_2574267

**Bacterial and virus strains**		

*E. coli* DH10B	Thermo Fisher Scientific	EC0113

**Chemicals, peptides, and recombinant proteins**		

Pierce Protease Inhibitor Mini Tablets, EDTA-free	Thermo Scientific	A32955
Protease inhibitor cocktail	Sigma-Aldrich	P8340
PhosSTOP	Roche	04 906 837 001
TWEEN 20	Merck	P9416
Bovine Serum Albumin (BSA)	ITW Reagents	A6588
Sodium chloride (NaCl)	Merck	CAS # 7647-14-5
Dithiothreitol (DTT)	AppliChem	A1694
Ethylenediaminetetraacetic acid dipotassium salt dihydrate (EDTA)	Sigma-Aldrich	CAS # 25102-12-9
Nonidet P40 (NP-40)	AppliChem	A1694
Tris base	AppliChem	A1086
Paraformaldehyde	ThermoFisher Scientific	43368.9M
Protein Assay Dye Reagent Concentrate	BioRad	5000006
Mevastatin (Mev)	Alfa Aesar	J61357.MB
2-Bromohexadecanoic acid (2-BP)	Sigma	21604-1G
AMG510	Medchem express	HY-114277
AMG510	Selleckchem	S8830
ARS1620	Selleckchem	S8707
MRTX849	Selleckchem	S8884
Dimethyl sulfoxide (DMSO)	PanReac AppliChem	A3672
Dimethyl sulfoxide, hybri-max	Merck	D2650
Epidermal Growth Factor (EGF)	Sigma-Aldrich	E9644
G418	Roche	0472787001
Trypsin Gold, mass spectrometry grade	Promega	V5280
L-Lysine 4 (4,4,5,5,D_4_) [H2NCH2(CD2) 2CH2CH(NH2)CO2H.2HCl]	Cambridge Isotope Laboratories	DLM-2640-0.5
L-Arginine 6 (^13^C_6_) [H2N∗C(=NH)NH (∗CH2)3∗CH(NH2)∗COOH·HCl]	Cambridge Isotope Laboratories	CLM-2265-H-0.5
L-Lysine 8 (^13^C_6_,^15^N_2_) [H2∗N(∗CH2) 4∗CH∗NH2)∗COOH·2HCl]	Cambridge Isotope Laboratories	CNLM-291-H-0.5
L-Arginine 10 (^13^C_6_,^15^N_4_) [H2∗N∗C(=∗NH)∗NH (∗CH2)3∗CH(∗NH2)∗COOH·HCl]	Cambridge Isotope Laboratories	CNLM-539-H-PK
Hoechst dye	ThermoFisher Scientific	H1399
VECTASHIELD Mounting Medium	Vector Laboratories	H-1000.10
Dulbecco’s phosphate-buffered saline (DPBS) (1 ×)	ThermoFisher Scientific	14040091
Dulbecco’s phosphate-buffered saline (DPBS) (10 ×), no calcium, no magnesium	ThermoFisher Scientific	14200067
Dulbecco’s modified Eagle’s medium (DMEM)	ThermoFisher Scientific	41965039
DMEM, high glucose, GlutaMAX Supplement, pyruvate	ThermoFisher Scientific	31966021
Opti-MEM Reduced Serum Medium	ThermoFisher Scientific	31985047
Trypsin EDTA (0.05%)	ThermoFisher Scientific	25300054
Trypsin EDTA (0.05%), no phenol red	ThermoFisher Scientific	15400054
Fetal bovine serum (FBS)	ThermoFisher Scientific	A5256701
Fetal bovine serum (FBS)	ThermoFisher Scientific	10437010
Fetal bovine serum (FBS), dialyzed	BioSera	FB-1001D
Horse serum (HS)	ThermoFisher Scientific	16050130
Penicillin-Streptomycin (10,000 U/mL)	ThermoFisher Scientific	15140122
L-glutamine (200 mM)	ThermoFisher Scientific	25030024

**Critical commercial assays**		

Gateway LR Clonase II enzyme mix	ThermoFisher Scientific	11791020
Coelenterazine 400a; 2,8-Dibenzyl-6-phenyl-imidazo[1,2*a*]pyrazin-3-(7H)-one; DeepBlueC	Gold Biotechnology	C-320-1
jetPRIME transfection reagent	Polyplus	101000046
RNAiMAX transfection reagent	Invitrogen by ThermoFisher Scientific	13778150
GFP-Trap Agarose	Chromotek	gta-20
Pierce Streptavidin magnetic beads	Thermo Scientific	88817
Pierce BCA protein concentration assay	Thermo Scientific	A55864
MycoAlert Plus	Lonza	#LT07-710

**Deposited data**		

The mass spectrometry proteomics data have been deposited to the ProteomeXchange Consortium via the PRIDE partner repository.	This paper	PXD067415

**Experimental models: Cell lines**		

Human cell line, HEK293-EBNA (HEK)	Prof. Florian M. Wurm, EPFL, and from ATCC	CRL-10852 RRID: CVCL_6974
Human cell line, HEK293T	Horizon Discovery	N/A
Human cell line, HEK293T (FLIM-FRET)	gift	N/A
Human cell line, HEK293	ATCC	CRL-1573
Human cell line, HEK293-TurboID	This paper	N/A
Mouse cell line, C2C12	ATCC	CRL-1772 RRID:CVCL_0188

**Oligonucleotides**		

*See*Data S2*for full list of siRNAs used in this study.*		

**Recombinant DNA**		

pDest305-CMV-GFP2-K-Ras4bG12V (mutated P01116-2)	Okutachi S. et al.^24^	N/A
pDest305-CMV-RLuc8-K-Ras4bG12V (mutated P01116-2)	Okutachi S. et al.^24^	N/A
pEF-C-RBD-GFP2	Steffen C.L. et al.^12^	N/A
pDest305-CMV-GFP2-H-RasG12V (mutated P01112-1)	Okutachi S. et al.^24^	N/A
pDest305-CMV-RLuc8-H-RasG12V(mutated P01112-1)	Okutachi S. et al.^24^	N/A
CMV51p>NanoLuc-Hs.KRAS4bG12V	RAS-Initiative FNLCR	R733-M03-305
pDest305-CMV-mNeonGreen-KRasG12V	This paper	N/A
pDest305-CMV-mNeonGreen-KRasG12C	This paper	N/A
pDest312-CMV-mNeonGreen	This paper	N/A
pDest305-CMV-NanoLuc-H-RasG12V	Steffen C.L. et al.^12^	N/A
pDest305-CMV-mNeonGreen-H-RasG12V	Steffen C.L. et al.^12^	N/A
pcDNA3.1(−)	ThermoFisher Scientific	V79520
pDEST-305	Addgene	161895
pDEST-312	Addgene	161897
C413-E36_CMV promoter	Addgene	162927
C453-E04_CMV promoter	Addgene	162973
pmCherry-C1	Clontech Laboratories Inc., Mountain View, CA, USA	N/A
pmCherry-C3-HA-K-RasG12V	Abankwa, D. et al.^9^	N/A
pmEGFP-K-RasG12V	Abankwa, D. et al.^9^	N/A
pDONR221_mNeonGreen	GeneCust (Boynes, France)	N/A
Hs. K-Ras4bG12V (mutated P01116-2)	RAS mutant collection V2.0	RAS-Initiative Addgene, #83132
Hs. K-Ras4B G12C (mutated P01116-2)	RAS mutant collection V2.0	RAS-Initiative Addgene, #83130
pDest312-CMV-GFP2	Okutachi S. et al.^24^	N/A
pDest305-CMV-GFP2-C-Raf	Steffen C.L. et al.^12^	N/A
pEF-APLP2-SNAP (aa 1–763)	GeneCust (Boynes, France)	N/A
pEF-SNAP-SPRY2 (aa 1–315)	GeneCust (Boynes, France)	N/A
pEF-SNAP-N-SPRY2 (aa 1–160)	GeneCust (Boynes, France)	N/A
pEF-SNAP-C-SPRY2 (aa 161–315)	GeneCust (Boynes, France)	N/A
pDest305-CMV-mNeonGreen-SPRY4	GeneCust (Boynes, France)	N/A
pDest305-CMV-mNeonGreen-SPRY2	GeneCust (Boynes, France)	N/A
pDest305-CMV-mNeonGreen-N-SPRY2	GeneCust (Boynes, France)	N/A
pDest305-CMV-mNeonGreen-C-SPRY2	GeneCust (Boynes, France)	N/A
pDest305-CMV-NanoLuc-SPRY2	GeneCust (Boynes, France)	N/A
pDest305-CMV-mNeonGreen-mutSPRY2 (K187A/Y191A/T298A)	GeneCust (Boynes, France)	N/A
pDest305-CMV-mNeonGreen-mutC-SPRY2 (K187A/Y191A/T298A)	GeneCust (Boynes, France)	N/A
pDest305-CMV-NanoLuc-mutK-RasG12V (Q25A/R40A/Y41A)	GeneCust (Boynes, France)	N/A
pDest305-CMV-mNeonGreen-C-Raf-RBD	This paper	N/A
pDest305-CMV-mCherry-C-Raf	This paper	N/A
pcDNA3.1-CMV-HA-TurboID	Genscript	N/A
pcDNA3.1-CMV-HA-TurboID-tK	Genscript	N/A
pcDNA3.1-CMV-HA-TurboID-K-Ras4B	Genscript	N/A
pcDNA3.1-CMV-HA-TurboID-K-Ras4B G12C	Genscript	N/A
pcDNA3.1-PGK-HA-TurboID-K-Ras4B G12C	Genscript	N/A
pcDNA3.1-CMV-HA-TurboID-K-Ras4B G12V	Genscript	N/A

**Software and algorithms**		

ChimeraX v1.9	Resource for Biocomputing, Visualization and Informatics (RBVI) at UCSF Meng et al.^71^	https://www.cgl.ucsf.edu/chimerax/
GraphPad Prism v10.4.2	GraphPad by Dotmatics,	https://www.graphpad.com/
ImageStudioLite v5.2.5	LI-COR Biosciences	https://www.licor.com/bio/odyssey-clx/
CLARIOstar software	BMG LABTECH	N/A
GuavaSoft v4.0	Cytek Biosciences	N/A
Fiji ImageJ2 v2.16.0	Rueden et al.^72^	N/A
NIS-Elements Imaging Software v6.10.01	Nikon	N/A
MaxQuant v1.6.17	Max-Planck Institute	https://maxquant.org/
Perseus v1.6.15.0 and v2.0.9.0	Max-Planck Institute	https://maxquant.org/
Thermo Fisher Instrument Control Software v3.3	Thermo Fisher Scientific	https://www.thermofisher.com
Dionex Chromatography Mass Link (DCMSLink) v2.12/2.14	Thermo Fisher Scientific	https://www.thermofisher.com
Thermo Fisher Exactive Series Control Software v2.12	Thermo Fisher Scientific	https://www.thermofisher.com

**Other**		

Trans-Blot Turbo RTA Transfer Kit, Nitrocellulose	BioRad	1704271
Nitrocellulose membrane (0.45 μm)	Amersham Protran	10600002
Genie Blotter	Idea Scientific	4017
Amersam ECL Full-Range Rainbow Marker	Cytiva	RPN800E
Page Ruler Prestained Protein Ladder	Thermo Scientific	26616
NuPAGE Bis-Tris Mini Protein Gels, 4–12%, 1.0–1.5 mm	Thermo Fisher Scientific	NP0321
NuPAGE Bis-Tris Mini Protein Gels, 4–12%, 1.0–1.5 mm	Thermo Fisher Scientific	NP0336
NuPAGE Bis-Tris Midi Protein Gels, 4 to 12%, 1.0 mm	Thermo Fisher Scientific	WG1402
NuPAGE MOPS SDS Running Buffer (20 X)	Thermo Fisher Scientific	NP0001
Mini-PROTEAN TGX TM Precast Protein Gels 4–20%	BioRad	4561094
Mini-PROTEAN TGX TM Precast Protein Gels 4–20%	BioRad	4561093
10% Criterion XT-Bis-Tris Protein Gel, 18 well	BioRad	3450112
Glass coverslips 1.5H	Carl Roth	LH22.1
Pierce C-18 Spin Columns	Thermo Scientific	89870
CLARIOstar Plus Microplate Reader	BMG LABTECH	https://www.bmglabtech.com/en/clariostar-plus/
Odyssey CLx Infrared Imaging System	LI-COR Biosciences	https://www.licor.com/bio/odyssey-clx/
Inverted microscope AXIO Observer D1	Zeiss	https://www.zeiss.com/microscopy/en/products/light-microscopes/widefield-microscopes/axio-observer-for-life-science-research.html#features
Lambert Instruments FLIM Attachment (LIFA)	Lambert Instruments	https://www.lambertinstruments.com/lifa#lifa-introduction
Eclipse Ti2-E spinning disk confocal microscope	Nikon	https://www.microscope.healthcare.nikon.com
iXon Ultra 888 EMCCD camera	Andor, Oxford Instruments	https://andor.oxinst.com/
APO 60×/1.40 Ph3 DM oil immersion objective	Nikon	https://www.microscope.healthcare.nikon.com
Guava easyCyte 6HT 2L flow cytometer	Cytek Biosciences	0500–4007
UltiMate 3000 RSLCnano system	Thermo Fisher Scientific	https://www.thermofisher.com
Thermo Orbitrap Fusion Mass spectrometer	Thermo Fisher Scientific	https://www.thermofisher.com
Q Exactive HF Hybrid Quadrupole-Orbitrap Mass Spectrometer	Thermo Fisher Scientific	https://www.thermofisher.com

### Experimental model and study participant details

#### Cell lines and cell culture

BRET experiments were performed in the HEK293-EBNA cell line gifted by Prof. Florian M. Wurm (EPFL Switzerland) or purchased from ATCC (CRL-10852). Cells were cultured in Dulbecco’s modified Eagle Medium (DMEM) (ThermoFisher Scientific, #41965039) supplemented with ∼9% (v/v) Fetal Bovine Serum (FBS) (ThermoFisher Scientific, A5256701), 2 mM L-Glutamine (ThermoFisher Scientific, #25030024) and penicillin-streptomycin (ThermoFisher Scientific, #15140122) 10,000 units/mL, in T75 (Greiner, #658175) or T175 (Greiner, #660175) flasks. Cells were cultured in a humidified incubator maintained at 37°C and 5% CO_2_ and routinely passaged 2–3 times per week.

HEK293T cells were a gift from Horizon Discovery, HEK293 were purchased from ATCC (CRL-1573). HEK293-TurboID stable cell lines were generated by transfection and selection of stable transformants with G418. Single clones were not isolated. For SILAC labeling, cells were grown for five passages in DMEM supplemented with 10% dialyzed FBS (BioSera, #FB-1001D) containing light, medium or heavy-labelled arginine and lysine (Cambridge Isoptope Laboratories). Labeling was confirmed to be ≥95% using liquid chromatography mass spectrometry (LC-MS) prior to experiments. All HEK293 derived cell lines are of female origin.

The mouse muscle C2C12 cell line (female mouse origin) was purchased from ATCC (CRL-1772) and cultured in DMEM supplemented with ∼9% (v/v) FBS, 2 mM L- and penicillin-streptomycin 10,000 units/mL (high serum medium). Cells were cultured in a humidified incubator maintained at 37°C and 5% CO_2_ and passaged at about 50% confluency. To induce differentiation, the medium was exchanged with DMEM supplemented with ∼2% horse serum (ThermoFisher Scientific, #16050130), 2 mM L-glutamine and penicillin/streptomycin at 10,000 units/mL (low serum medium).

Cells were routinely tested for mycoplasma contamination using MycoAlert Plus mycoplasma Detection kit (Lonza, #LT07-710). All purchased cell lines are ATCC authenticated.

### Method details

#### Expression constructs

TurboID constructs were generated by Genscript in pcDNA3 vector under the control of either CMV or PGK promoter. The design included an N-Terminal HA-tag, followed by the linker GSGSGGSG, then the TurboID sequence separated from the K-Ras sequence by linker GGSGSGSG.^73^

Multi-site Gateway cloning was used to generate plasmids encoding mNeonGreen-tagged (mNG) C-Raf-RBD, K-RasG12V and K-RasG12C as well as the control vector containing only mNG, and mCherry-C-Raf, as described in the previous studies by us.^24^^,^^25^^,^^74^ The entry clone plasmid encoding mNG was synthetised by GeneCust. In brief, three entry clones, encoding the CMV promoter, the tag and the gene of interest or a stuffer sequence for the control vector, with compatible LR recombination sites, were recombined with a destination vector, pDest-305 or pDest-312, using the Gateway LR Clonase II enzyme mix (Thermo Fisher Scientific, #11791020). The Gateway reaction mix was then transformed into the ccdB-sensitive *Escherichia coli* strain DH10B (Thermo Fisher Scientific, #EC0113), and positive clones were selected using ampicillin. All final clones were verified using Sanger sequencing by Eurofins.

#### Western blotting

For SDS-PAGE analysis of TurboID experiments, NuPage 4–12% Bis-Tris gels (ThermoFisher, #NP0321, #NP0336, #WG1402) and MOPS running buffer (ThermoFisher, #NP0001) were used. The gels were transferred to a nitrocellulose membrane (Amersham Protran 0.45 μm, #10600002) using Genie Blotter (Idea Scientific, #4017) and the membranes were blocked either in 5% milk powder (Marvel) or 5% bovine serum albumin (BSA) both diluted in Tris-buffered saline (TBS) supplemented with 0.1% TWEEN 20. Primary antibodies were diluted in a blocking buffer and incubated at 4°C overnight, secondary antibodies were diluted 1:15,000 in TBS with 0.1% TWEEN 20 and 5% milk and incubated for 1 h at room temperature (20°C–25°C). Streptavidin-800CW conjugate was diluted at 1:5,000 and incubated as per secondary antibodies. Amersham ECL Full-Range Rainbow Marker (Cytiva, #RPN800E) was used as a protein size reference.

For the remaining experiments, Mini-PROTEAN TGX TM Precast Protein Gels 4–20% (BioRad, #4561094 and #4561093) were used. Page Ruler Prestained Protein Ladder (Thermo Scientific, #26616) was used as a protein size reference. For analysis by Western blot, the samples were transferred to a 0.2 μm nitrocellulose membrane using Trans-Blot Turbo RTA Midi-size Nitrocellulose Transfer Kit (BioRad, #1704271). The membranes were rinsed with water and then blocked in TBS supplemented with 0.2% Tween 20 and 2% BSA (ITW Reagents, #A6588, 0100) for 1 h at room temperature (20°C–25°C). Primary antibodies were diluted in a blocking buffer and were incubated overnight at 4°C. All secondary antibodies were diluted 1:10,000 in a blocking buffer and were incubated 1 h at room temperature (20°C–25°C).

Membranes were visualised using an Odyssey CLx Imaging System. Quantitation of bands was performed using ImageStudioLite software (v5.2.5) and graphs were plotted using Graph Pad Prism 10.4.2.

#### TurboID experiments

Cells were grown in SILAC media for mass spectrometry experiments (“Light” = unlabelled; “medium” = Lysine 4 [4,4,5,5 – D_4_], Arginine 6 [^13^C_6_]; “heavy” = Lysine 8 [^13^C_6_, ^15^N_2_], Arginine 10 [^13^C_6_, ^15^N_4_]). For the first experiment (K-Ras proximity), HEK293T cells were transfected using the calcium phosphate method. Media was changed 1 h prior to transfection. For a 15 cm dish, 17.5 μg DNA was mixed with 75 μL 2.5M CaCl_2_ and diluted to 700 μL using 0.1 × TE (1 mM Tris-HCl pH7.6, 0.1 mM EDTA) before adding dropwise to 700 μL 2 × HBS (50 mM HEPES pH7.05, 140 mM NaCl, 1.5 mM Na_2_PO_4_ while vortexing. Complexes were allowed to form for 3 min at 37°C with shaking at 600 rpm, before adding dropwise to cells. After 3 h incubation at 37°C, cells were washed and media was changed, and cells were incubated for approximately 24 h before treatment for experiments. TurboID pull-downs were performed as described elsewhere.^73^ Transfected cells were treated with 50 μM biotin (Sigma-Aldrich, #B4501) or DMSO for 10 min at 37°C where indicated, before washing three times in ice-cold PBS and lysis in RIPA buffer (50 mM Tris pH 7.5, 150 mM NaCl, 0.1% SDS, 0.5% sodium deoxycholate, 1% Triton X-100) supplemented with 50 mM NaF, PhosSTOP (Roche, #04 906 837 001), 2 mM sodium orthovanadate, 0.1 mM phenylmethylsulfonyl fluoride (PMSF) and mammalian protease inhibitors (Sigma-Aldrich, #P8340). Lysates were sonicated, clarified by centrifugation and the concentration determined by Pierce BCA protein assay (Thermo Scientific, #A55864). Lysates were mixed 1:1:1 (light:medium:heavy) and 3 mg pulled down using 250 μL streptavidin magnetic beads (Thermo Scientific, #88817). Beads were washed with 2 × RIPA, 1 × 1 M KCl, 1 × 0.1 M Na_2_CO_3_, 1 × 2 M Urea/10 mM Tris-HCl pH 8.0, 2 × RIPA, 1 × 50 mM Tris pH 7.5, 2 × 2 M Urea/50 mM Tris pH 7.5. For digestion, beads were incubated with 400 ng Trypsin Gold (Promega, #V5280) in 2 M urea/Tris pH 7.5 for 1 h at 25°C. The digest solution was removed from the beads and the digestion allowed to continue overnight before reduction, alkylation and dehydration.

For the second experiment (G12Ci-sensitive proximity), SILAC-labelled HEK293 cells stably expressing TurboID constructs were treated with K-RasG12C inhibitors or vehicle for 3 h and 50 min at 37°C where indicated, prior to 10 min of biotin treatment. Inhibitor concentrations were 1 μM AMG510, 10 μM ARS1620 or 3 μM MRTX849. Lysis was as above, then a total of 900 μg equally mixed (1:1:1) lysate was pulled down with 75 μL strepatavidin beads as before. Beads were boiled at 95°C in 3 × protein loading buffer (165 mM Tris pH 6.8, 17% glycerol, 5% SDS, 0.045% DTT, 0.6% bromophenol blue) supplemented with 2 mM biotin and 20 mM freshly added DTT, then loaded onto an SDS-PAGE gel. Peptides were yielded by in-gel trypsin digest. All LC-MS samples were desalted using C18 spin columns prior to LC-MS analysis (Thermo Scientific, #89870).

TurboID pulldowns for Western blotting were performed as per mass spectrometry experiments 1 or 2, but without SILAC labeling.

#### LC-MS analysis

In experiment 1, the Ras proximal proteins were analyzed by Warwick Scientific Service, University of Warwick. Tryptic peptides from three biological replicates were analyzed using an UltiMate 3000 RSLCnano system coupled to a Thermo Orbitrap Fusion (Q-OT-qIT, Thermo Scientific). Peptides were loaded onto a trapping column (Acclaim PepMap μ-precolumn, 300 μm × 5 mm, 5 μm packing material, 100 Å; ThermoFisher Scientific) in 4% acetonitrile/0.1% formic acid, then eluted onto the analytical column (Acclaim PepMap RSLC 75 μm × 50 cm^2^ μm 100 Å; ThermoFisher Scientific) by increasing from 4% acetonitrile/0.1% formic acid to 25% acetonitrile/0.1% formic acid over 36 min, then to 35% acetonitrile/0.1% formic acid over 10 min, and to 90% acetonitrile/0.1% formic acid over 3 min, followed by a 10 min re-equilibration at 4% acetonitrile/0.1% formic acid. Eluting peptides were ionised using an electrospray source and analyzed on a Thermo Orbitrap Fusion (Q-OT-qIT, Thermo Scientific). MS survey scans from 375 to 1575 *m*/*z* were acquired at 120K resolution (at 200 *m*/*z*) with a 50% normalised automatic gain control (AGC) target and a max injection time of 150 ms. Precursor ions with charge state of 2–6 were selected for MS/MS by isolation at 1.2 Th. Higher-energy collisional dissociation (HCD) fragmentation was performed with normalised collision energy of 33, and rapid scan MS analysis in the ion trap. The MS^2^ was set to 50% normalised AGC target and the maximum injection time was 150 ms. The dynamic exclusion duration was set to 45 s with a 10 ppm tolerance. Monoisotopic precursor selection was turned on. The instrument was run in top speed mode with 2 s cycles.

In experiment 2, the G12Ci sensitive proteome was analyzed in the Center for Proteomics Research, University of Liverpool. Peptides from three biological replicates were analyzed using an UltiMat 3000 RSLCnano system coupled to a Q Exactive HF Hybrid Quadrupole-Orbitrap Mass Spectrometer (ThermoFisher Scientific). Peptides were loaded onto a trapping column (Acclaim PepMap 100 C18, 75 μm × 2 cm, 3 μm packing material, 100 Å; ThermoFisher Scientific) using 0.1% trifluoroacetic acid, 2% acetonitrile in water at a flow rate of 12 μL min^−1^ for 7 min. Peptides were eluted onto an analytical column (EASY-Spray PepMap RSLC C18, 75 μm × 50 cm, 2 μm packing material, 100 Å) at 30°C using a linear gradient of 90 min rising from 3% acetonitrile/0.1% formic acid to 40% acetonitrile/0.1% formic acid at a flow rate of 300 nL min^−1^. The column was then washed with 79% acetonitrile/0.1% formic acid for 5 min, and re-equilibrated to starting conditions. The nano-liquid chromatograph was operated under the control of Dionex Chromatography MS Link 2.14. The nano-electrospray ionisation source was operated in positive polarity under the control of QExactive HF Tune, with a spray voltage of 1.8 kV and a capillary temperature of 250°C. Full MS survey scans between m/z 350-2,000 were acquired at a mass resolution of 60,000 (full width at half maximum at m/z 200) with AGC target set to 3e^6^ and a maximum injection time of 100 ms. The 16 most intense precursor ions with charge states of 2–5 were selected for MS^2^ with an isolation window of 2 m/z units. HCD was performed to fragment the selected precursor ions using a normalised collision energy of 30%. Product ion spectra were recorded between m/z 200-2,000 at a mass resolution of 30,000 (full width at half maximum at m/z 200). For MS^2^ the AGC target was set to 1e^5^ and the maximum injection time was 45 ms. Dynamic exclusion was set to 30 s.

#### LC-MS data analysis

The raw data were searched using MaxQuant version 1.6.17 against a human Swissprot and tREMBL database (experiment 1) or version 2.2.0 against a human Swissprot database (experiment 2). For the database search, peptides were generated from a tryptic digestion with up to two missed cleavages, carbamidomethylation of cysteines as fixed modification. Oxidation of methionine, biotinylation of lysine and acetylation of the protein N-terminus were added as variable modifications. Further analysis of the data was performed using Excel and Perseus (v1.6.15.0 for experiment 1, v2.0.9.0 for experiment 2). Protein IDs that matched the reverse or known contaminants list were excluded. To allow for ratio generation in Excel, a nominal value of 1,000 was added to all intensities. Protein IDs that were present in at least two repeats for one construct were retained. To refine a shortlist for continuation to BRET-assays, mean ratios (+biotin/-biotin) of intensities for protein IDs from all three repeats of experiment 1 were generated and only hits increased by at least 2-fold compared to the -biotin condition in full-length TurboID-KRAS constructs but not TurboID-tK or TurboID alone were retained. This list was combined with common hits from previously published Ras BioID experiments and controls to generate a shortlist of proteins of interest for inclusion in BRET-based proximity assays.^15^^,^^16^^,^^17^

For experiment 2, a proximity list was defined as any proteins enriched at least 2-fold in the TurboID-G12C plus biotin condition vs. TurboID-G12C minus biotin. Of this group, G12Ci-sensitive hits were defined as any that were less enriched in TurboID-G12C plus G12Ci plus biotin, vs. TurboID-G12C plus biotin. Only hits with at least two values returned for at least two of any of the G12Ci conditions were retained.

#### GFP-trap mediated pull-downs

HEK293-EBNA cells were plated in a 10 cm dish scale so that for transfection they would be 50–70% confluent (2 × 10^6^ cells per dish for next day transfection). For each sample, one 10 cm dish was plated. For transfection, 5 μg of each DNA to be transfected was diluted in 500 μL jetPRIME buffer. For single-plasmid transfections, jetPRIME was used at 3:1 ratio (3 μL of jetPRIME per 1 μg of DNA transfected) and for plasmid co-transfection at 2:1 ratio (2 μL of jetPRIME per 1 μg of DNA). After approximately 24 h, the growth medium was removed and the cells were rinsed gently twice with cold PBS (DPBS, Gibco, #14040091). Next, 500 μL of the lysis buffer (2 mM EDTA, 50 mM Tris pH 7.5, 150 mM NaCl, 0.5% NP-40, Pierce Protease Inhibitor Tablet EDTA-free (Thermo Scientific, #A32955), PhosSTOP (Roche, #04 906 837 001, and 1 mM DTT) was applied to each dish, the cells were scraped, transferred to an Eppendorf tube and incubated on ice for 30 min. The samples were mixed by gently inverting 3–4 times. The lysate was cleared by centrifuging for 15 min at 4°C and ∼16,500 × *g*. Cleared lysate was transferred to a clean Eppendorf tube and subjected to GFP-Trap pull-down (GFP-Trap Agarose, ChromoTek #gta-20). For pull-downs, 25 μL bead slurry per 10 cm cell dish were considered. The required volume of bead slurry was pipetted to an Eppendorf tube and rinsed three times with equilibrating buffer (10 mM Tris pH 7.5, 150 mM NaCl, 0.5 mM EDTA). The beads were finally re-suspended in the equilibrating buffer to have 1:1 bead:buffer ratio. Pull-downs were conducted for 3 h at room temperature (20°C–25°C) with samples in gentle rotation. Next, the beads were pelleted by centrifuging at 4°C and 800 × *g* for 1 min, supernatant was removed and the beads were rinsed with 1 mL washing buffer (2 mM EDTA, 50 mM Tris pH 7.5, 150 mM NaCl, 0.5% NP-40, 1 mM DTT), at 4°C for 15 min on a rotating wheel. The washing step was repeated for total of four times. The bound material was released from the beads by adding 25 μL of 2 × SDS-PAGE sample buffer and incubating at 95°C for 5 min. Supernatant was recovered by centrifuging at 800 × *g* for 1 min and transferred to a clean Eppendorf tube. The samples were resolved on 4–20% SDS-PAGE gels in Tris-Glycine buffer and further analyzed by Western blotting. For each sample, 2% of total lysate was loaded as input control.

#### Bioluminescence resonance energy transfer (BRET) assays

For screening the effect of TurboID hits siRNA-mediated knockdown in HEK293-EBNA cells, we employed BRET-assays with RLuc8-fused BRET donor, GFP2-fused BRET acceptor and coelenterazine 400a as the substrate.^27^ A CLARIOstar plate reader (BMG Labtech) was used for measurements. The cells were plated in a 12-well cell culture plate (Greiner Bio-One, #655180). The following day, the cells were transfected with siRNA at the final concentration of 100 nM using Lipofectamine RNAiMAX diluted in Opti-MEM (ThermoFisher Scientific, #31985047), while the cells were left in complete DMEM. After ∼24 h the siRNA-containing medium was replaced with fresh DMEM and ∼1 μg BRET-sensor constructs was transfected per well at the donor:acceptor plasmid ratio of 1:15 (65 ng of the BRET-donor plasmid) and using 3 μL jetPRIME transfection reagent. The pcDNA3.1(−) control plasmid was used to top up the DNA amount.

The following day, the control samples were treated with 10 μM mevastatin, or DMSO as vehicle control at 0.1% (v/v) for 16–18 h. About 48 h after transfection of BRET-sensors, the cells were plated in a white flat bottom 96-well plate (Thermo Fisher Scientific, #236108) and BRET measurements were taken.

First, the fluorescence intensity of GFP2 was measured (*λ*_excitation_ 405 ± 10 nm and *λ*_emission_ 515 ± 10 nm). This signal is directly proportional to the expression of the BRET-acceptor (RFU). Next, coelenterazine 400a was prepared by diluting in PBS to 100 μM concentration, and 10 μL was dispensed to each well, thus making its final concentration in the well 10 μM. BRET was recorded simultaneously at *λ*_emission_ 410 ± 40 nm (RLU) and 515 ± 15 nm (BRET signal). RLU is directly proportional to the expression of the BRET-donor. BRET ratio was calculated as the ratio of BRET signal/RLU and the final BRET ratio was obtained by subtracting the background BRET ratio obtained from cells expressing only BRET-donor plasmid:

$$\text{BRET}\;\text{ratio}=\frac{BRET\;channel\;\left(donor+acceptor\right)}{donor\;channel\;\left(donor+acceptor\right)}-\frac{BRET\;channel\;\left(donor\;only\right)}{donor\;channel\;\left(donor\;only\right)}$$

The percentage modulation on BRET (ΔBRET) is calculated as:

ΔBRET = |100−[100∗(Mean BRET ratio of sample/Mean BRET ratio of scrRNA)]|. A negative sign is added if the gene decreases the BRET ratio. Heatmaps were generated in Prism from calculated averaged BRET ratios across all biological repeats.

Interaction of SPRY2 fragments with K-RasG12V, mutK-RasG12V, K-RasG12C, H-RasG12V, SPRY2 and SPRY4 was assessed by BRET titration experiments using a modified pair of BRET-sensors, with nanoLuc (nL)-fused BRET-donor and mNeonGreen (mNG)-fused BRET acceptor. The concentration of BRET-donor plasmid was kept constant (25 ng) and BRET-acceptor plasmid was titrated to 1:40 donor:acceptor plasmid ratio. The pcDNA3.1(−) plasmid was used to top-up plasmid amount in each well to ∼1 μg and 3 μL of jetPRIME transfection reagent were used per well.

Clariostar settings were adjusted as follows: for fluorescence intensity (RFU), *λ*_excitation_ 485 ± 10 nm and *λ*_emission_ 535 ± 10 nm, and for BRET measurements, *λ*_emission_ 460 ± 25 nm (RLU) and 535 ± 25 nm (BRET signal). Coelenterazine a was used at 2.9 μM final concentration. Compounds were prepared at the indicated concentrations by diluting in DMEM and 1 mL of the mix was added to each well, for the total of 18–24 h, with DMSO 0.05% or 0.01% (v/v) as vehicle control.

The BRET ratio was plotted against acceptor:donor (A/D) plasmid ratio and was fitted using one-phase association fit in Prism. The BRETtop corresponds to the highest BRET ratio reached at acceptor:donor = 40:1 plasmid ratio and is given as mean with standard error (SEM).

For the dose-response BRET assays, the donor and acceptor plasmids were transfected at the fixed ratio of 1:8, with 25 ng donor-plasmid, and the modulator plasmid was titrated at increasing amounts up to 775 ng transfected. The pcDNA3.1(−) plasmid was used to top-up plasmid amount in each well to ∼1 μg and 3 μL of jetPRIME transfection reagent were used per well.

BRET ratios were calculated as described above and were normalised against sample without the modulator being set to one. The data were plotted in GraphPad Prism10.4.2 using non-linear fit with plateau constrained to 0. The BRET ratio at the highest amount of modulator plasmid transfected was compared to the BRET ratio reached for 5 μM mevastatin-treated sample.

For the dose-response BRET with AMG510, the donor and acceptor plasmids were transfected at the fixed ratio of 1:40, with 25 ng donor-plasmid. The cells were treated with the compound at indicated concentrations, or DMSO vehicle control for about 24 h. The data were plotted in GraphPad Prism10.4.2 using non-linear fit [Inhibitor] vs. response equation, from which IC_50_ was calculated.

#### Efficiency of APLP2 and SPRY2 knock-down

150,000 HEK293-EBNA cells were seeded per well in a 12-well cell culture plate (Greiner Bio-One #655180). siRNA transfection was done as described above, using 100 nM final siRNA concentration. The samples were collected 72 h after silencing on 100 μL of lysis buffer consisting of 150 mM NaCl, 50 mM Tris-HCl pH 7.4, 1% (v/v) NP40, 0.1% (v/v) SDS, 1 mM EDTA 2 mM DTT, 1 × PhosSTOP (Roche, #04 906 837 001), 1 × Pierce Protease Inhibitor Tablet EDTA-free (Thermo Scientific, #A32955). The cells were lysed on ice for 30 min with occasional vortexing, then cleared by centrifugation for 15 min at 4°C and ∼16,500 × *g*. Lysates were quantified using Bradford assay (Protein Assay Dye Reagent Concentrate, Bio-Rad #5000006), 25 μg of total protein was resolved on 10% SDS-PAGE (10% Criterion XT-Bis-Tris Protein Gel,18 well, Bio-Rad #3450112) and further analyzed by Western blotting.

#### MAPK-signalling Western blotting

400,000 cells were seeded per well in a 12-well cell culture plate (Greiner Bio-One, #655180). The cells were transfected with 1 μg plasmid per well using 3 μL jetPrime transfection reagent. The following day, the growth medium was removed and the cells were rinsed gently twice with DMEM serum-free medium and placed in 1 mL of serum-free medium overnight (∼18 h). The following day, the cells were treated with epidermal growth factor (EGF, Sigma-Aldrich #E9644) at 200 ng/mL for 10 min, then the plate was placed on ice, the medium removed and the cells rinsed twice in cold PBS and collected on 100 μL of lysis buffer consisting of 150 mM NaCl, 50 mM Tris-HCl pH 7.4, 1% (v/v) NP40, 0.1% (v/v) SDS, 1 mM EDTA, 2 mM DTT, 1 × PhosSTOP (Roche, #04 906 837 001), 1 × Pierce Protease Inhibitor Tablet EDTA-free (Thermo Scientific, #A32955). The cells were lysed on ice for 30 min with occasional vortexing, then cleared by centrifugation at by centrifuging for 15 min at 4°C and ∼16,500 × *g*. Lysates were quantified using Bradford assay (Protein Assay Dye Reagent Concentrate, Bio-Rad #5000006) and 25 μg of total protein was resolved on 4–20% SDS-PAGE and further analyzed by Western blotting.

Images were visualised using Licor software and quantified using Fiji ImageJ.

For dual normalisation, signal from each individual antibody (pERK, ERK, SNAP) was normalised against its loading control (GAPDH) to correct for loading differences. Then normalised pERK and ERK signals were divided by GAPDH-normalised SNAP-tag signal to correct for differences in expression of the SNAP-tagged protein and finally the pERK/ERK ratio was calculated. Value for SNAP-tag alone was set to one and other samples were calculated accordingly. Values were plotted in GraphPad Prism showing mean ± SEM and analyzed using unpaired *t* test with Welch's correction.

#### FLIM-FRET measurements

A total of 80,000 HEK293T cells were seeded in 12-well plates. Each well contained a sterilised 16-mm coverslip and 1 mL of medium. The next day, the cells were transfected using jetPRIME at 1:1 ratio (1 μL of jetPRIME for 1 μg of DNA). For donor-only samples, the cells were transfected with 500 ng of pmEGFP-tagged K-RasG12V plasmid. For K-RasG12V nanoclustering-FRET, the cells were transfected with a total of 1 μg of pmEGFP/pmCherry-K-RasG12V, at a donor: acceptor plasmid ratio of 1:3. For gene silencing experiments, the cells were co-transfected with 100 nM siRNA along with the FRET plasmids using jetPRIME. After 4 h, the media was changed. After 48 h of transfection, the cells were fixed in 4% paraformaldehyde for 10–15 min. The cells were then mounted on slides using Mowiol 4–88. The lifetime of the mGFP donor was measured per cell using a fluorescence microscope (Zeiss AXIO Observer D1) with a lifetime imaging attachment from Lambert Instruments. The FRET efficiency (E) was calculated using the formula: E = (1 − τDA/τD) × 100%, where τDA is the lifetime of an FRET condition averaged across all acquired samples, and τD is the averaged lifetime of the donor-only sample.

#### Confocal microscopy

HEK293-EBNA cells were seeded at 350,000 cells in 2 mL of complete DMEM onto Glass coverslips 1.5H (Carl Roth, Karlsruhe, Germany, #LH22.1) in 6-well plates (Greiner Bio-One, #657160). The following day, the cells were transfected with 0.25 μg of plasmid diluted in 200 μL jetPRIME buffer and 2 μL jetPRIME transfection reagent. For double transfection when the total plasmid amount was 0.5 μg, the same amount of buffer and jetPRIME reagent was used. Compound treatments were done 6 h after transfection by recovering the medium from each well in Eppendorf tubes and diluting the compounds to the indicated concentrations. After 18 h of treatment, the medium was removed and the cells were rinsed with PBS. Next, the cells were fixed using 4% paraformaldehyde (ThermoFisher Scientific, #43368.9M) in PBS and left to incubate for 10 min at 20°C–25°C. The fixing solution was removed and the coverslips were covered with a 1:2,000 dilution of Hoechst dye (ThermoFisher Scientific, #H1399) for 5 min. After removal of Hoechst dye, the cells were washed twice with PBS. The coverslips were then mounted using VECTASHIELD Mounting Medium (Vector Laboratories, #H-1000-10) and left to dry. The slides were observed on a Nikon Eclipse Ti2-E spinning disk confocal microscope with an iXon Ultra 888 EMCCD camera (Andor, Oxford Instruments) using a plan APO 60×/1.40 Ph3 DM oil immersion objective (Nikon, Belgium) and NIS-Elements Imaging Software (Nikon, Version 6.10.01). The mNeonGreen-label was detected using 488 nm excitation and detected using EGFP emission settings (535/20 band-pass filter). The mCherry-label was detected using 561 nm excitation and a 560/40 band-pass filter for signal detection. Images were analyzed using Fiji ImageJ2 (v2.16.0).

#### Flow cytometry-based C2C12 cell differentiation assay

The differentiation assay was performed on a flow cytometer Guava easyCyte HT-2L flow cytometer as described by us previously.^52^^,^^53^ In brief, C2C12 cells were seeded in 6-well plates at a density of 20,000 cells in 2 mL. The next day, cells were transiently transfected at 60% confluency using jetPRIME at a 3:1 ratio (3 μL of jetPRIME per 1 μg of DNA) with 2 μg of mNG-SPRY2, mNG-N-SPRY2 or mNG-C-SPRY2 diluted in 200 μL of jetPRIME buffer. The medium was replaced by fresh medium 4 h later. 24 h after transfection, the wells were switched to low serum DMEM containing ∼2% horse serum. The medium was changed every day for three days. Then, cells were harvested by trypsinisation for 5 min and centrifuged at 500 × *g* for 5 min. The cell pellet was fixed with 4% paraformaldehyde (ThermoFischer Scientific, #43368.9M) in PBS for 10 min. After washing with PBS, the cells were permeabilised with 0.5% Triton X-100 in PBS for 10 min. Next, the cells were washed with PBS containing 0.05% Tween 20 (PBST) and immunolabelled with eFluor 660-conjugated anti-myosin 4 (myosin heavy chain, MyHC) antibody (ThermoFisher Scientific, #50-6503-82), at 1:100 dilution in PBST for 1 h at 4°C. The cells were then centrifuged at 500 × *g* for 5 min and resuspended in PBST for flow cytometric analysis. Non-labelled, mNG-only expressing and MyHC-positive cells were used to establish gates and detection channel settings. The mNG-variants were detected by 488 nm excitation and the Grn-B (525/30) band-pass filter. MyHC immunolabelled with eFluor 600-conjugated antibody was detected by 640 nm excitation and the Red-R (664/20) band-pass filter. Typically, >1,000 cells were analyzed in the final target gate per experimental repeat. Intact cells were quantified for the expression of mNG in the mNG-low bin (up to 10-fold above background fluorescence) and MyHC using the ‘Quad Stat plot’ feature on the GuavaSoft 4.0 software.

### Quantification and statistical analysis

Data were analyzed using GraphPad Prism10.4.2 software. The number of independent biological repeats (N) is indicated in the figure legends. Plotted are means with standard error (SEM) unless stated otherwise.

The statistical evaluations of the Western blotting quantifications were calculated using unpaired t test with Welch's correction. All BRETtop values were compared using One-Way Brown-Forsythe and Welch ANOVA tests with Dunnett's T3 correction for multiple comparisons, or unpaired t test with Welch's correction if only two groups were compared, using GraphPad Prism. Other statistical tests are comparisons to the control and are described in the legends. A p-value of <0.05 was considered statistically significant and p values are annotated in the figures.

## Supplemental information

Document S1. Figures S1–S7

Data S1. Excel file with genes identified in K-Ras proximal proteome analysis, related to Figure 1 Sheets 1 and 2 list gene names of hits from the K-Ras proximal proteome (experiment 1) and G12Ci sensitive proteome (experiment 2) experiments. Mean log2 ratios of biotinylated/non-biotinylated (for proximity) or biotinylated+G12Ci/biotinylated (for G12Ci-sensitive) from N = 3 independent biological repeats. Sheet 3 shows common hits from our meta-analysis of other published Ras proximal proteome studies. Identification of a protein with the provided gene ID is annotated with a 1 if detected or 0 if not detected.

Data S2. List of siRNAs used in this study, related to key resources table
