# Supplementary material for: Proteomics- and BRET- screens identify SPRY2 as a Ras effector that impacts its membrane organization
Source: iScience. 2025 Nov 7;28(12):113974. doi: 10.1016/j.isci.2025.113974 (PMC12682140; doi:10.1016/j.isci.2025.113974)
Supplement: Document S1. Figures S1–S7 [file mmc1.pdf]

iScience, Volume *28*

Supplemental information

Proteomics- and BRET- screens identify SPRY2 as a Ras effector that impacts its membrane organization

Karolina Pavic, Fiona Elizabeth Hood, Carla Jane Duval, Ganesh babu Manoharan, Christina Laurini, Farid Ahmad Siddiqui, Stephanie Puy Lam Mo, Ian Andrew Prior, and Daniel Kwaku Abankwa

Supplemental Information

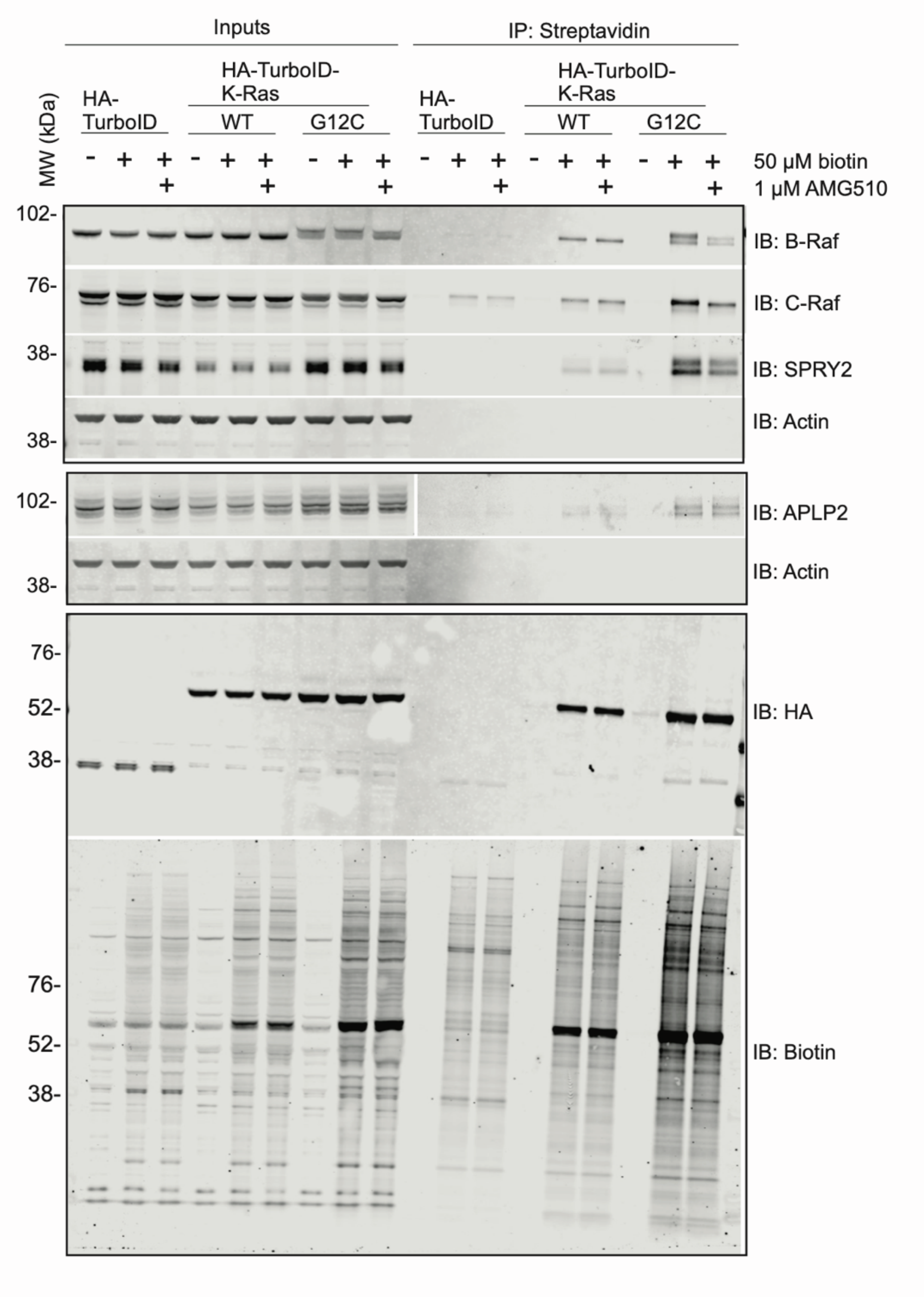

Figure S1. Western blotting data confirming TurboID mediated biotinylation and G12Ci-sensitivity. Related to Figure 1. Representative Western blots of the K-Ras proximal labelling and its activation dependence for a subset of hits showing successful biotinylation, streptavidin enrichment, K-RasG12C inhibitor (G12Ci) sensitivity of selected hits. Input versus post-streptavidin enrichment of biotinylated proximal proteins using the same TurboID experiment configuration employed for mass spectrometry analysis (N = 3). For the APLP2 blot, input and IP are from different exposures indicated by white space.

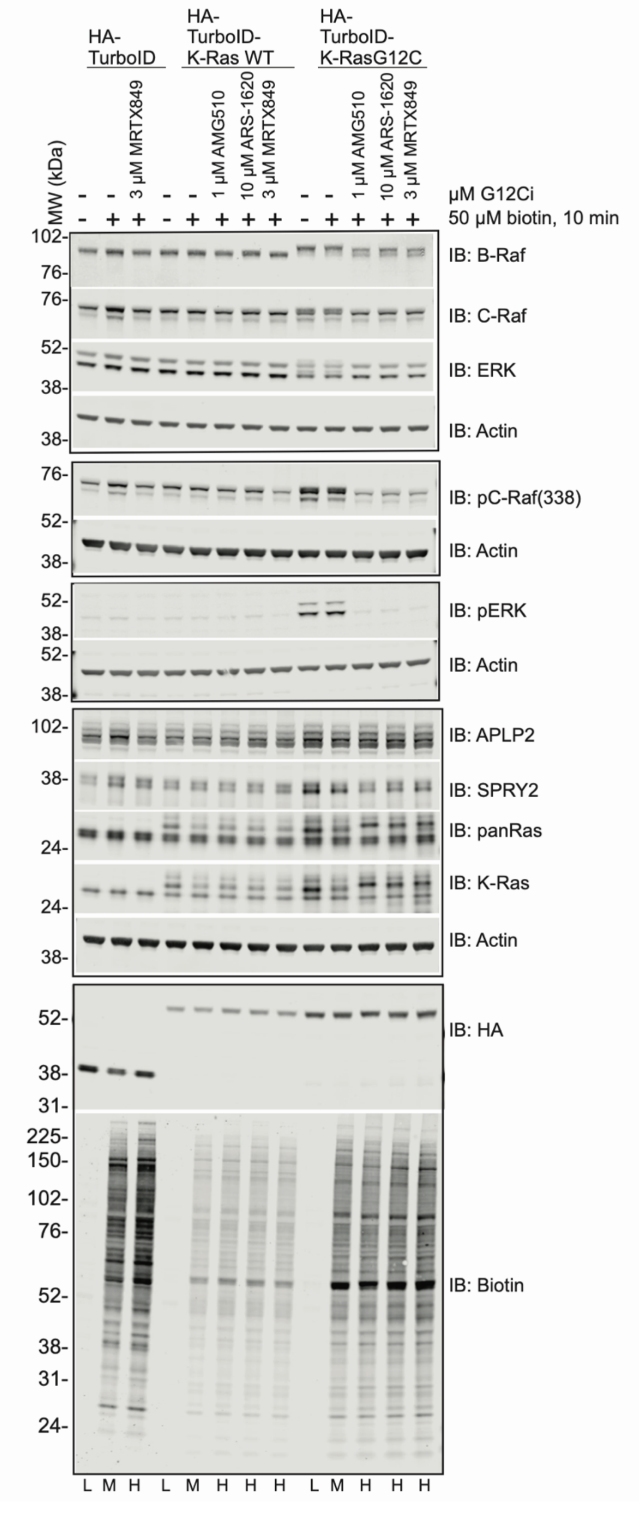

Figure S2. Western blotting data confirming G12Ci-sensitive TurboID biotinylation. Related to Figure 1. Activation-dependent changes with a panel of K-Ras inhibitors in stable HEK293-TurboID cell lines. Responses to three different covalent K-RasG12C inhibitors (G12Ci) are shown. The lysates come from one of the total of N = 3 repeats of the TurboID streptavidin pull-downs for mass spectrometry analysis of SILAC-labelled cells.

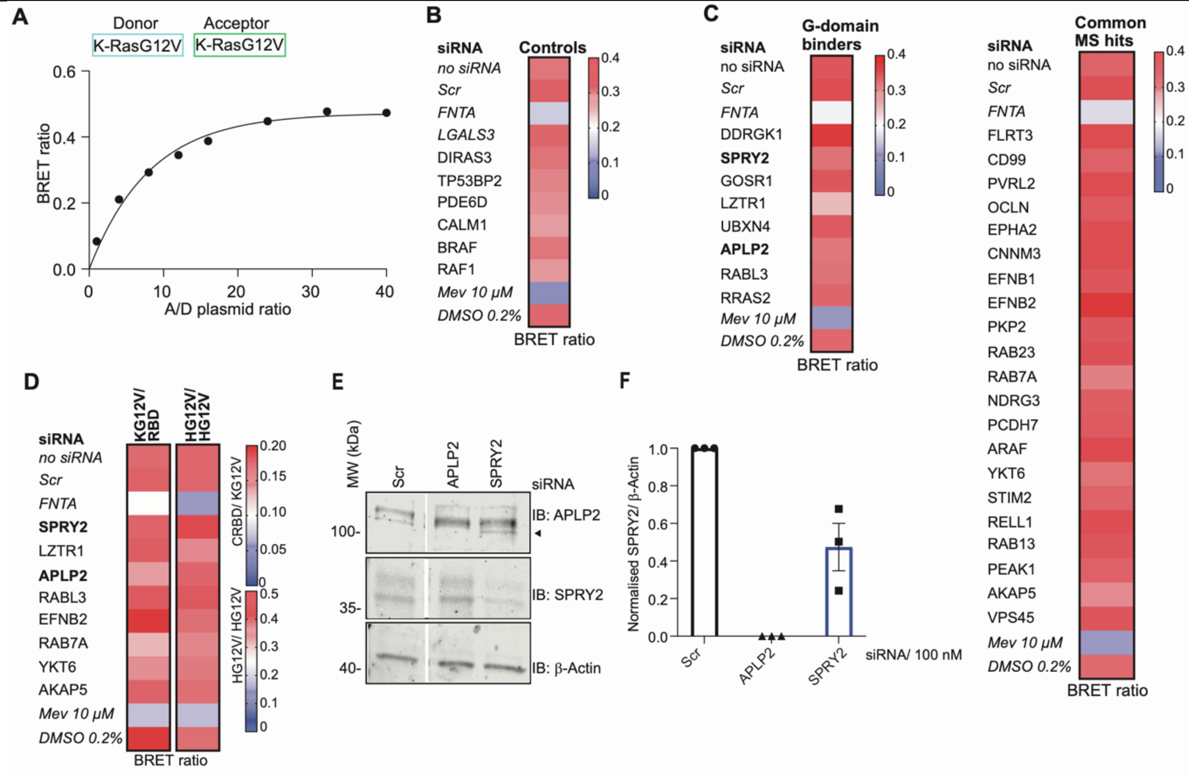

Figure S3. BRET-screen heatmaps. Related to Figure 1. (**A**) BRET-titration curve of Rluc8-K-RasG12V/ GFP2-K-RasG12V in HEK-cells from N = 3 independent biological repeats. The pseudo-linear regime of this curve around 1:15 A/D plasmid ratio was selected for the subsequent screening (**B,C**) Heatmaps of BRET ratio values after knockdown of the indicated genes in the BRET-assay to measure K-RasG12V membrane organisation in HEK cells (donor:acceptor plasmid ratio = 1:15). The control set of genes (B) and our selected TurboID hits combined with hits from our meta-analysis (C) were examined. Data show means from N ≥ 4 independent biological repeats. (**D**) Heatmaps of BRET ratio values after knockdown of the indicated genes in BRET-assays to measure K-RasG12V/ C-Raf-RBD interaction (KG12V/ RBD, donor:acceptor plasmid ratio = 1:15) and H-RasG12V membrane organisation (HG12V/ HG12V, donor:acceptor plasmid ratio = 1:15) in HEK cells. Data show means from N = 4 independent biological repeats. (**E,F**) Representative blots showing knockdown efficiency of APLP2 (arrowhead) and SPRY2 in HEK cells with white space indicating non-consecutive lanes, (E) with quantified means ± SEM from N = 3 biological repeats (F).

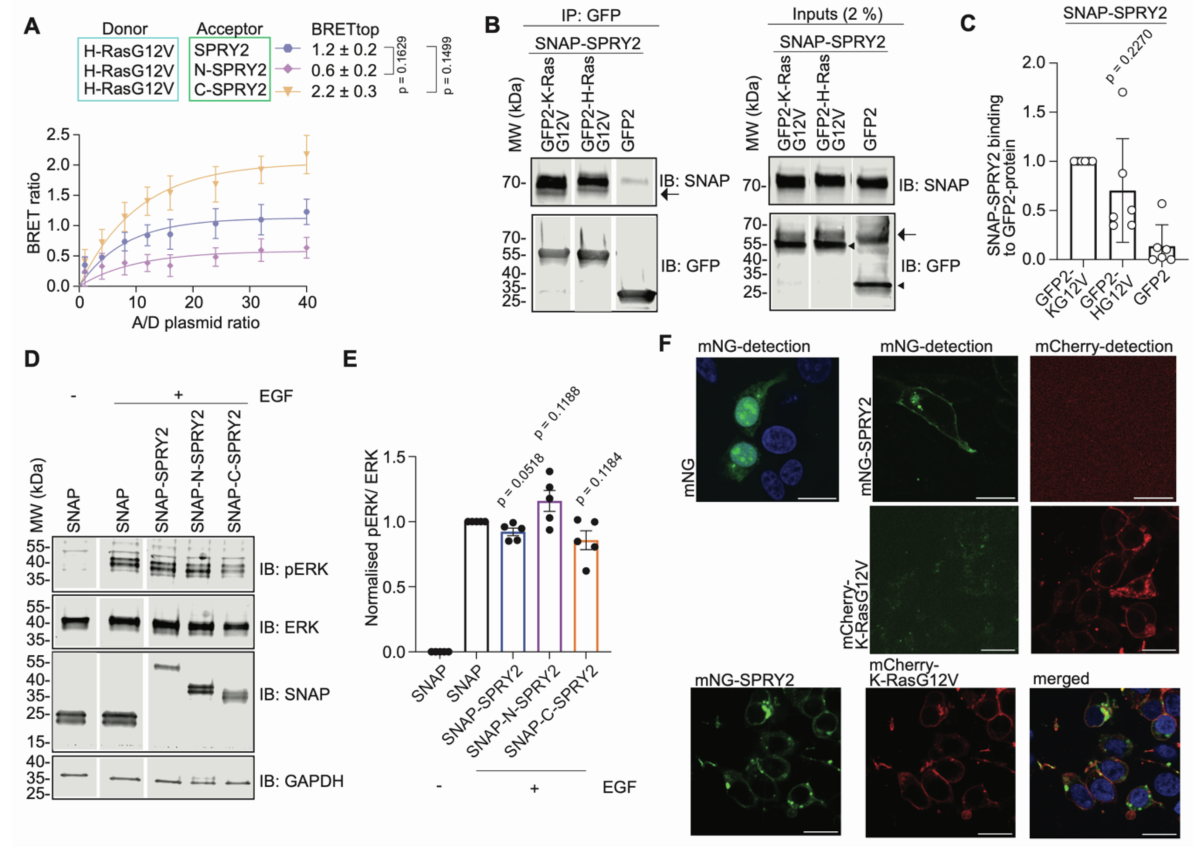

Figure S4. SPRY2 binding and localisation data. Related to Figure 3. (**A**) BRET-titration curves of the nL-H-RasG12V interaction with mNG-tagged SPRY2 fragments in HEK cells from N = 3 biological repeats. One-Way Brown-Forsythe and Welch ANOVA tests with Dunnett´s T3 correction for multiple comparisons. (**B,C**) Representative blots of GFP-Trap pull-downs (left) of SNAP-SPRY2 from HEK lysates (right) using GFP2-K-RasG12V, GFP2-H-RasG12V or GFP2 (control) as indicated (B) with quantified means ± SEM from N = 6 biological repeats of pull-down data analysed using unpaired t test with Welch´s correction (C). Arrowheads mark specific bands of expressed constructs and arrows mark non-specific bands. White space indicates non-consecutive lanes (B). (**D,E**) Representative immunoblot data of lysates from HEK cells transfected with indicated SNAP-tagged SPRY2-constructs and stimulated with EGF (D) with quantified means ± SEM (E) from N = 5 biological repeats analysed using unpaired t test with Welch´s correction. White space indicates non-consecutive lanes (D). (**F**) Confocal imaging showing expression of mNG without fused protein for reference (top left), imaging crosstalk controls (top right), and co-localisation of mCherry-K-RasG12V with mNG-SPRY2 in HEK cells (bottom). Scale bar = 20 µm.

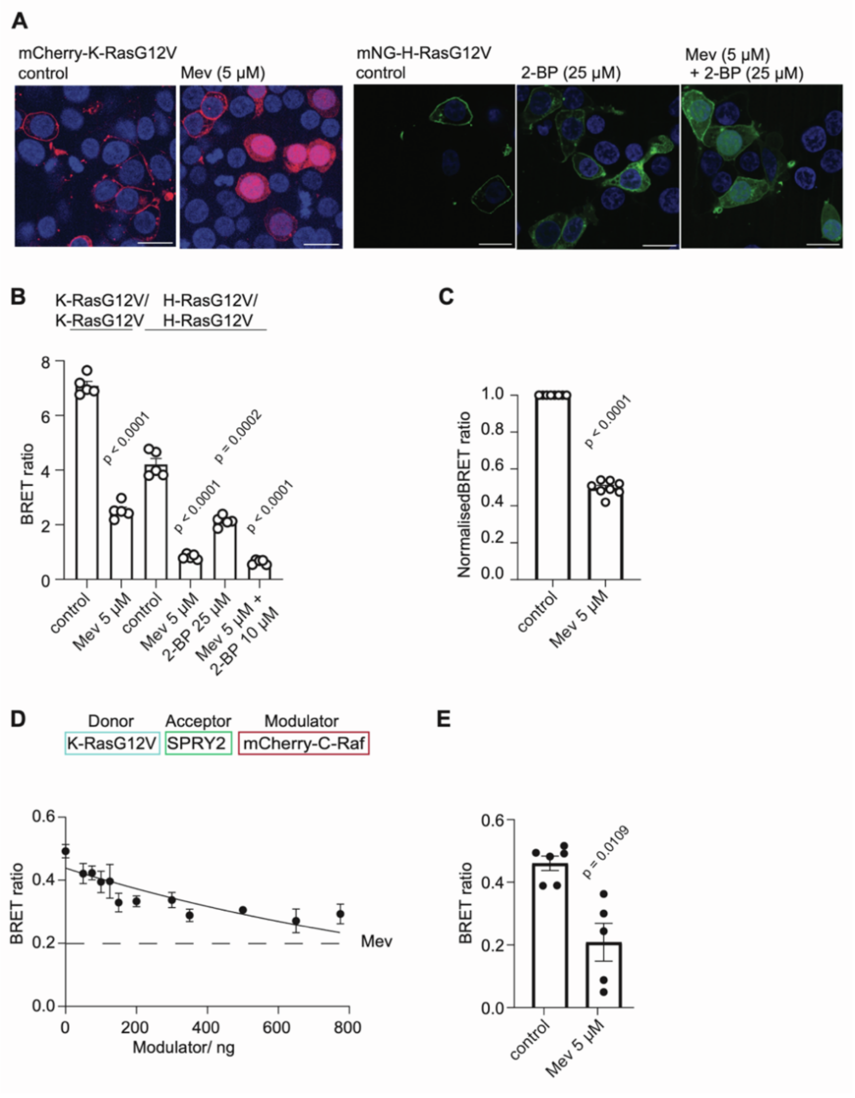

Figure S5. Sensitivity of SPRY2 localisation to lipidation inhibitors and competition BRET data. Related to Figure 4. (**A**) Confocal imaging showing changes in subcellular distribution of mCherry-K-RasG12V (left) or mNG-H-RasG12V (right) in HEK cells following treatment with indicated lipidation inhibitors mevastatin (Mev) and/ or 2-bromopalmitate (2-BP). Scale bar = 20 µm. (**B**) Effect of mevastatin (Mev) and/ or 2-bromopalmitate (2-BP) on interaction BRET of nL-K-RasG12V/ mNG-K-RasG12V (donor:acceptor plasmid ratio = 1:8) and nL-H-Ras-G12V/ mNeonGreen-H-RasG12V (donor:acceptor plasmid ratio = 1:5) in HEK cells from N = 5 independent biological repeats. (**C**) Effect of 5 µM mevastatin (Mev) treatment on nL-K-RasG12V/ mNG-C-Raf-RBD BRET in HEK cells (donor:acceptor plasmid ratio = 1:8) from N = 8 independent biological repeats analysed using Welch´s t test. (**D,E**) Dose-dependent effect of mCherry-C-Raf expression on nanoLuc-K-RasG12V/ mNG-SPRY2 BRET in HEK cells from N = 3 - 6 biological repeats (C), with dashed line indicating response to 5 µM mevastatin (Mev) treatment (D) from N ≥ 5 biological repeats analysed using Welch´s t test.

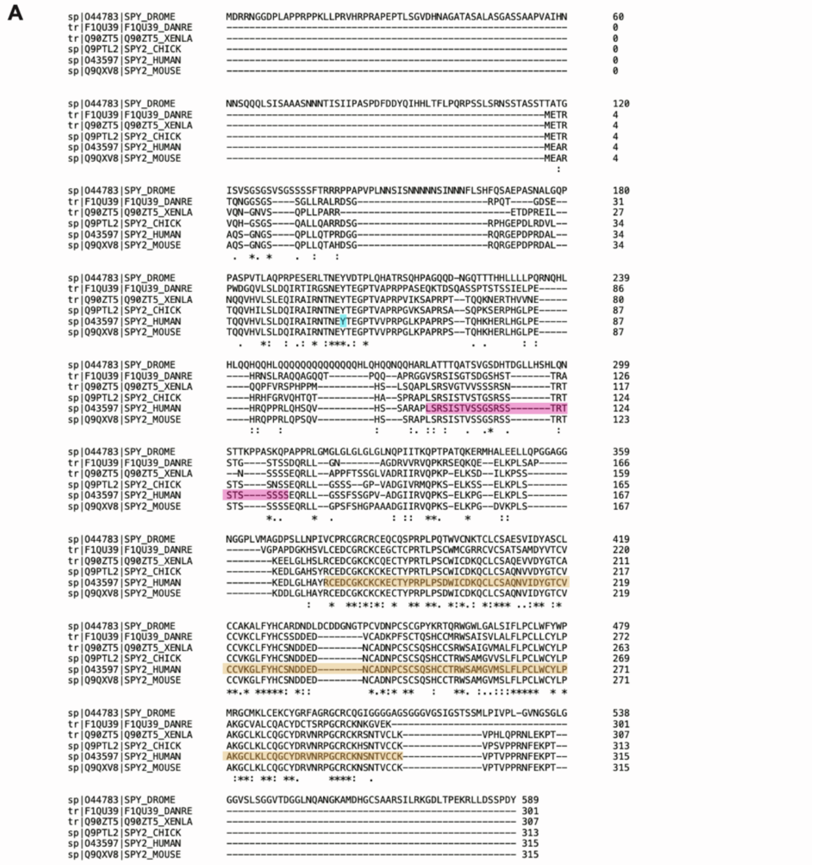

B

K-Ras Human	SPRY2 Human	Mouse	Chick	Frog	Fish	Fly
I36	L43	L	L	L	L	Q
I36	I46	I	I	I	I	P
Y64	I46	I	I	I	I	P
D33	A48	A	A	A	T	S
P34	I49	I	I	I	I	E
Y32	N53	N	N	N	N	N
L120	Y55	Y	Y	Y	Y	Y
S39	K187	K	K	Q	E	E
I55	K187	K	K	Q	E	E
**Y40**	**K187**	K	K	Q	E	E
**R41**	**Y191**	Y	Y	Y	C	S
E175	E234	E	E	E	E	D
**Q25**	**T298**	T	T	T	V	G

C

SPRY2 Human	K-Ras Human	Mouse	Chick	Frog	Fish	Fly
L43	I36	I	I	I	I	I
I46	I36	I	I	I	I	I
I46	Y64	Y	Y	Y	Y	Y
A48	D33	D	D	D	D	D
I49	P34	P	P	P	P	P
N53	Y32	Y	Y	Y	Y	Y
Y55	L120	L	L	L	L	L
K187	S39	S	S	S	S	S
K187	I55	I	I	I	I	I
**K187**	**Y40**	Y	Y	Y	Y	Y
**Y191**	**R41**	R	R	R	R	R
E234	E175	E	G	G	G	R
**T298**	**Q25**	Q	Q	Q	Q	Q

D

K-Ras	SPRY2	SPRY1	SPRY3	SPRY4
I36	L43	L	I	I
I36	I46	I	L	V
Y64	I46	I	L	V
D33	A48	A	S	T
P34	I49	I	T	S
Y32	N53	N	N	N
L120	Y55	Y	Y	Y
S39	K187	G	V	K
I55	K187	G	V	K
**Y40**	**K187**	G	V	K
**R41**	**Y191**	A	A	S
E175	E234	E	E	D
**Q25**	**T298**	T	N	S

Figure S6. SPRY protein sequence comparisons. Related to Figure 5. (**A**) Multiple sequence alignment of SPRY2 protein sequences obtained from UniProt across several species using Clustal Omega (v1.2.4). Residues of the cysteine rich domain (orange), serine rich motif (pink) and Tyr55 (blue) at the centre of the Cbl-TKB binding motif are highlighted in the sequence of human SPRY2. Protein IDs for SPRY2 are human- O43597, mouse- Q9QXV8, chick- Q9PTL2, frog- Q90ZT5, fish- F1QU39, fly- O44783. (**B,C**) Comparison for SPRY2 (B) and K-Ras (C) of the amino acid conservation across species of all K-Ras/ SPRY2 interface residues predicted by AlphaFold3 to have a distance below 3 Å. Protein IDs for SPRY2 are given in (A). Protein IDs for K-Ras are human-P01116, mouse- P32883, chick- A0A8V0YNE2, frog- Q05147, fish- Q6AZA4, fly- P08646. (**D**) Comparison of the amino acid sequence conservation across human SPRY paralogs of residues as in (B,C). Protein IDs are SPRY2- O43597, SPRY1- O43609, SPRY3- O43610 and SPRY4- Q9C004.

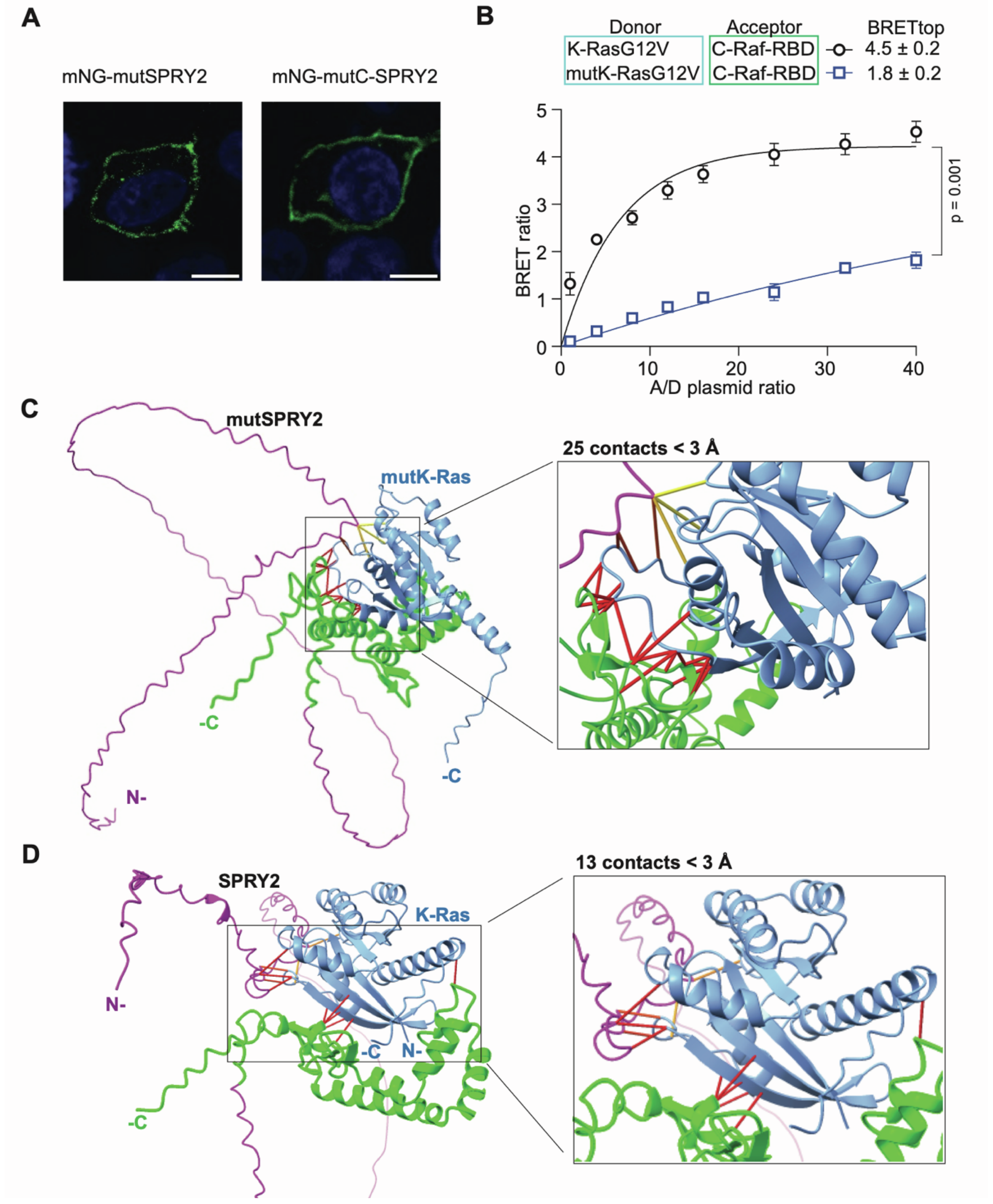

Figure S7. SPRY localisation, interaction and structural data. Related to Figure 5. (**A**) Confocal imaging showing subcellular localisation of mNG-tagged mutSPRY2 and mutC-SPRY2 constructs in HEK cells. Scale bar = 10 µm. (**B**) BRET-titration curves of the nL-K-RasG12V interaction with mNG-C-Raf-RBD in HEK cells from N = 4 biological repeats analysed using Welch´s t test. (**C,D**) AlphaFold 3 prediction of the mutK-Ras/ mutSPRY2 (C) or the K-Ras/ SPRY2 (D) interaction, with zoom-in showing contacts within 3 Å distance (red and yellow straight lines).
