# Supplementary material for: Proteomics- and BRET- screens identify SPRY2 as a Ras effector that impacts its membrane organization
Source: iScience. 2025 Nov 7;28(12):113974. doi: 10.1016/j.isci.2025.113974 (PMC12682140; doi:10.1016/j.isci.2025.113974)
Supplement: Data S2. List of siRNAs used in this study, related to key resources table [file mmc3.pdf]

Data S2: List of siRNAs used in this study. Related to Key Resources Table.

Oligonucleotides		
ON-TARGETplus Non-targeting Pool	Dharmacon	D-001810-10-20
Human *FNTA* siRNA	Qiagen	SI02661995
Human *LGALS3* siRNA	Santa Cruz Biotechnology	sc-155994
Human *DIRAS3* siRNA	Dharmacon	L-008660-00-0005
Human *TP53BP2* siRNA	Dharmacon	L-004753-00-0005
Human *PDE6D* siRNA	Dharmacon	L-004310-00-0005
Human *CALM1* siRNA	Qiagen	SI02224222
Human *BRAF* siRNA	Dharmacon	L-003460-00-0005
Human *RAF1* siRNA	Ambion by life technologies	103578
Human *DDRGK1* siRNA	Ambion by life technologies	30568
Human *SPRY2* siRNA	Ambion by life technologies	114582
Human *GOSR1* siRNA	Ambion by life technologies	111531
Human *LZTR1* siRNA	Ambion by life technologies	139656
Human *UBXN4* siRNA	Ambion by life technologies	s23239
Human *APLP2* siRNA	Ambion by life technologies	s1463
Human *RABL3* siRNA	Ambion by life technologies	148210

Human *RRAS2* siRNA	Ambion by life technologies	120510
Human *FLRT3* siRNA ON-TARGETplus SMARTpool	Dharmacon	L-010090-01-0005
Human *CD99* siRNA ON-TARGETplus SMARTpool	Dharmacon	L-016245-00-0005
Human *PVRL2* siRNA ON-TARGETplus SMARTpool	Dharmacon	L-003699-00-0005
Human *OCLN* siRNA ON-TARGETplus SMARTpool	Dharmacon	L-187897-00-0005
Human *EPHA2* siRNA ON-TARGETplus SMARTpool	Dharmacon	L-003116-00-0005
Human *CNNM3* siRNA ON-TARGETplus SMARTpool	Dharmacon	L-019438-00-0005
Human *EFNB1* siRNA ON-TARGETplus SMARTpool	Dharmacon	L-003658-00-0005
Human *EFNB2* siRNA ON-TARGETplus SMARTpool	Dharmacon	L-003659-00-0005
Human *PKP2* siRNA ON-TARGETplus SMARTpool	Dharmacon	L-012692-00-0005
Human *RAB23* siRNA ON-TARGETplus SMARTpool	Dharmacon	L-009789-00-0005
Human *RAB7A* siRNA ON-TARGETplus SMARTpool	Dharmacon	L-010388-00-0005
Human *NDRG3* siRNA ON-TARGETplus SMARTpool	Dharmacon	L-013533-01-0005
Human *PCDH7* siRNA ON-TARGETplus SMARTpool	Dharmacon	L-008837-00-0005
Human *ARAF* siRNA ON-TARGETplus SMARTpool	Dharmacon	L-003563-00-0005
Human *YKT6* siRNA ON-TARGETplus SMARTpool	Dharmacon	L-019237-01-0005
Human *STIM2* siRNA ON-TARGETplus SMARTpool	Dharmacon	L-013166-01-0005
Human *RELL1* siRNA ON-TARGETplus SMARTpool	Dharmacon	L-184601-00-0005
Human *RAB13* siRNA ON-TARGETplus SMARTpool	Dharmacon	L-008389-00-0005
Human *PEAK1* siRNA ON-TARGETplus SMARTpool	Dharmacon	L-005339-00-0005
Human *AKAP5* siRNA ON-TARGETplus SMARTpool	Dharmacon	L-011954-00-0005
Human *VPS45* siRNA ON-TARGETplus SMARTpool	Dharmacon	L-013407-01-0005
